# High-Throughput Metabolomics Evaluate the Efficacy of Total Lignans From Acanthophanax Senticosus Stem Against Ovariectomized Osteoporosis Rat

**DOI:** 10.3389/fphar.2019.00553

**Published:** 2019-05-29

**Authors:** Ai-hua Zhang, Zhi-ming Ma, Hui Sun, Ying Zhang, Jian-hua Liu, Fang-fang Wu, Xi-jun Wang

**Affiliations:** ^1^National TCM Key Laboratory of Serum Pharmacochemistry, Laboratory of Metabolomics, Department of Pharmaceutical Analysis, National Chinmedomics Research Center, Sino-America Chinmedomics Technology Collaboration Center, Heilongjiang University of Chinese Medicine, Harbin, China; ^2^National Engineering Laboratory for the Development of Southwestern Endangered Medicinal Materials, Guangxi Botanical Garden of Medicinal Plant, Nanning, China

**Keywords:** metabolomics, biomarker, metabolic pathway, lignans, herbal medicine, efficacy, acanthophanax senticosus, ovariectomized osteoporosis

## Abstract

Postmenopausal osteoporosis (PMOP) is a common clinical illness in postmenopausal women, but there is no effective drug at present. Metabolomics approach was used to explore the potential biomarkers of PMOP and evaluate the efficacy and therapeutic targets of total lignans in the stem of Acanthophanax senticosus (ASSL) on the ovariectomized osteoporosis model rats. UPLC/MS and pattern recognition methods were used for serum metabolites discovery to illustrate the pathological mechanism of PMOP model rats, and then revealing the intervention effect of ASSL. The pattern recognition result showed that serum metabolic profiles of the sham operation group and the model group were clustered clearly, and 16 potential biomarkers were finally identified (7 in positive ion mode and 9 in negative ion mode), and they are involved in 15 related metabolic pathways. After oral administration of ASSL, 10 biomarkers were found to be significantly up-regulated and mainly regulated metabolic pathways include unsaturated fatty acid biosynthesis, linoleic acid metabolism, and arachidonic acid metabolism, primary bile acid synthesis, tyrosine metabolism, etc. Our study demonstrated that the ASSL could affect the endogenous metabolites related metabolic mechanism, provides a pharmacological basis of the ASSL for PMOP treatment.

## Introduction

Osteoporosis (OP) is a systemic bone disease characterized by bone mass reduction, bone strength reduction, and bone microstructure degeneration with the aging and aging of the human body, and it is also the main cause of high fracture incidence in recent years, which can lead to bone fragility and increased fracture risk (Wang et al., 2018; Watts, 2018). It has irreversible characteristics, and its early intervention methods have become a problem and focus of the whole society. It is divided into primary osteoporosis, secondary osteoporosis and idiopathic osteoporosis. Primary osteoporosis is divided into two categories, I -type primary osteoporosis is PMOP and type II osteoporosis is elderly osteoporosis; the former often occurs at 50 years old women in the left and right. After menopause, women's ovarian function declines, bone mass decreases, the rate of bone turnover in the body increases, and the rate of bone absorption exceeds the rate of bone formation, resulting in bone mass loss, bone density decrease and bone fragility increase, which is called PMOP (Horne et al., 2018). The animal model of postmenopausal osteoporosis (OP) can be established by classical castration surgery (Tella and Gallagher, 2014; Drake et al., 2015). At present, the combination of drugs and calcium therapy has a significant effect on senile osteoporosis, and estrogen combined with calcium therapy is better. Estrogen replacement therapy can increase estrogen levels in postmenopausal women, increase systemic bone mass and inhibit osteoclast activity, and slow bone metabolism, which can significantly reduce the incidence of fractures (Hassan et al., 2010). In recent years, some new drugs have also been used for the prevention and treatment of OP. However, early prevention and treatment are still difficult.

More and more Chinese herbal medicines have shown effective therapeutic effects on OP. *Acanthopanax senticosus* (AS) is derived from the rhizomes and stem of the *Acanthopanax senticosus Rupr. Et Maxim. Harms*, which was first published in < Shen Nong Ben Cao> (Huang et al., 2011). It has the functions of nourishing and strengthening, and contains a large number of chemical components, such as saponins, polysaccharides, lignins, flavonoids, organic acids, etc. (Hassan et al., 2010; Zhang et al., 2010b, 2016; Liu et al., 2012; Sun et al., 2014, 2016; Wang et al., 2017). In clinic, it play an excellent clinical effect in the cardiovascular system (Fujikawa et al., 1996, 2005). In addition, modern pharmacological studies have shown that AS also has anti-platelet aggregation, anti-thrombosis, anti-aging, anti-fatigue anti-inflammatory, and even anti-tumor effects (Zhang et al., 2010a). The AS stem is also medicinal part and has an estrogen-like anti-osteoporosis effect (Lee et al., 2004).

Metabolomics could explore the changes of all metabolites in living organisms after pathophysiological stimulation (Fiehn, 2002; Boudah et al., 2013). All metabolites were systematically analyzed by the high sensitivity and selectivity detection techniques (Sun et al., 2012; Wang et al., 2013b; Zhang et al., 2014a, 2015a; Yan et al., 2017), and metabolomics can qualitatively and quantitatively analyze endogenous small molecule metabolites in living organisms (Liu et al., 2017). UPLC/MS has been widely used in metabolite research practice because of its high sensitivity and high throughput characteristics (Dettmer et al., 2010; Zhang et al., 2011; Wang et al., 2015b). Multivariate analysis methods such as data processing and analysis techniques such as principal component analysis (PCA), partial least squares discriminant analysis (PLS-DA) and orthogonal partial least squares discriminant analysis (OPLS-DA) are widely used in metabolomics research (Song et al., 2017). In this study, the total lignans in the stem of *Acanthopanax senticosus* (ASSL) were used as the research object, and UPLC/MS technique was used to analyze all the metabolites and then illustrate the pathological mechanism, serum metabolomics method was used to explore the effect and identify potential therapeutic targets, provided a pharmacological basis of the ASSL for PMOP treatment.

## Experimental Methods

### Chemicals and Materials

Alkaline phosphatase kit and tartrate-resistant acid phosphatase kit were purchased from the Institute of Bioengineering (Nanjing, China); Osteocalcin kit was purchased from Northern Biotechnology Research Institute (Beijing, China); sodium chloride injection was acquired from Sanlian Pharmaceutical Co., Ltd. (Harbin, China); iodophor disinfectant was purchased from Xinruida Disinfectant Co., Ltd. (De zhou, China); chloral hydrate and sodium carboxymethyl cellulose were obtained from the Institute of Photosynthetic Fine Chemicals (Tianjin, China); penicillin sodium powder injection from Harbin Pharmaceutical Group Pharmaceutical Factory; acetonitrile, methanol, acetone were obtained from Merck (chromatographic grade, Merck, Germany); Neil Estrone was purchased from Vikchi Biotech Co., Ltd. (Sichuan, China); other reagents and chemicals used were of analytical grade. Identification and structural characterization of compounds in the ASS was shown in Table S1 and Figure S1. We also had determined the content of total lignans in the ASS (Orchard et al., 2013).

### Animal Handling Procedures and Drug Treatment

#### Model Preparation

Experimental Animals: Clean SD female rats weighing 260 ± 20 g were provided by the Experimental Animal Center of Heilongjiang University of Traditional Chinese Medicine. Pre-test animals were conditioned for one week in a well-ventilated, quiet environment, with free access to water and standard feed. One week later, rats were randomly divided into the model group (OVX), sham operation group (SHAM), Nysterious group (NYL, 1 mg/kg), and total lignans low dose group (ASSLL, 100 mg/kg), total lignans medium dose group (ASSLM, 200 mg/kg), total lignans high dose group (ASSLH, 400 mg/kg) with 8 rats per group. Except for the sham operation group, the remaining 50 rats were fasted for 12 h before surgery and anesthetized with 10% chloral hydrate (0.3 mL/100 g). The abdominal surface was fixed on the operating table, and the hair clipped range under the xiphoid process was 4 × 3 cm, and the surgical department was disinfected with iodine volts and 75% alcohol, respectively. The skin under the xiphoid process was 1.5 cm, and a 2 cm long, 2–2.5 cm deep incision is cut longitudinally along the white line of the abdomen to separate the abdominal muscles from peritoneum and the abdominal cavity was exposed. White adipose tissue is clearly visible at the incision. The fat layer is opened and the uterus is found. Gently pull out one side of the uterine horn, and see the pink morular-shaped ovary wrapped by fat at the end. Clamp the fallopian tube below the ovary with tissue forceps, and ligate the fallopian tube and its surrounding vascular adipose tissue with the sheep intestine. The ovaries are removed, and the uterine horn is sent back to the abdominal cavity, and the other side of the ovaries is removed by the same method. The muscle layer was first sutured with the gut, and the skin layer was sutured with a medical wire. Once again, the skin suture was disinfected with iodophor, and 50,000 units of penicillin sodium were injected into the abdominal cavity. The sham operation group took the same amount of adipose tissue around the ovary and did not perform oophorectomy. The rats in each group were given intraperitoneal injection of sodium cyclamate for 7 consecutive days at the rate of 50,000 units per day. After recovery of surgical wounds in each group, gavage was started on the 7th day. The sham operation group and the model group were given distilled water by gavage; the NYL group was intragastrically administered with nylestriol once a week, and distilled water was given by other time. The drug was administered at 8–10 a.m., and was administered continuously for 12 weeks. The dose was adjusted once a week according to the body weight.

#### Sample Collection and Determination

Rats in each group were fasted for 24 h after the last administration of the model for 12 weeks, and were free to drink water. After intraperitoneal injection of 10% chloral hydrate, 5 mL of blood samples were taken from the abdominal aorta, allowed to silence for 30 min, centrifuged at 3,000 rpm for 10 min at 4°C, and the supernatant fluid was removed and dispensed into a 1.5 mL centrifuge tube and frozen at −80°C. To be used for the determination of biochemical indicators of bone metabolism and blood metabolic profiles. Cut the back skin of the rat, separate the lumbar vertebrae (LV1-6) from the rat body, remove the muscle attached to the vertebrae, cut off the intervertebral disc and tendon, and remove the lumbar vertebrae (LV1-4) and lumbar vertebrae (LV5), respectively. Gauze of saline was wrapped and frozen at −20°C for the determination of the maximum load of bone density and bone biomechanical concave experiment. The uterus of each group was extracted, and the wet weight was weighed, according to the wet weight of the uterus (mg) /body weight (g) to calculate the uterus index.

#### Metabolomics Profiling

Serum samples were thawed at room temperature, 200 μL serum samples were taken, vortexed for 10 s, and add 800 μL equal proportion solution of methanol, acetonitrile, acetone to precipitate the protein, vortexed for 30 s, 4°C, 13,000 rpm, centrifuged for 20 min, and remove supernatant. Place in a 40°C water bath, blow dry with nitrogen, add 160 μL of methanol to the residue, vortex for 60 s, then add 40 μL of water, vortex for 60 s, centrifuge at 13,000 rpm for 10 min at 4°C, and take the supernatant through a 0.22 μm filter filtrate for UPLC analysis. Sample chromatographic conditions: ACQUITY UPLCTM HSS T3 (100 mm × 2.1 mm id, 1.8 μm); the mobile phase consisting of A (water and 0.1% formic acid) and B (acetonitrile and 0.1% formic acid) was analyzed at a column temperature of 40°C, a flow rate of 0.4 mL/min and a sample volume of 2 μL. The gradient was set as follows: 0–5 min: 99–65% A, 5–8 min: 65–45% A, 8–9.5 min: 45–30% A, 9.5–13 min: 30–0% A, maintaining 0% A for 2 min for equilibration of the column. Electrospray ionization source (ESI) was used for a full scan in positive and negative ion mode. The conditions were set as follows: ion spray voltage was set to 5.5 KV in positive ion mode and 4.0 KV in negative ion mode; ion source temperature was 600°C; the deconvolution voltage (DP) is 100 V and the collision energy (CE) is set to 35 eV. Nitrogen is the atomizing gas and the auxiliary gas, both are 55 psi and 65 psi in positive and negative ion modes, respectively, the blast gas is 35 psi.

#### Multivariate Statistical Analysis and Data Processing

UPLC-MS technology was used to analyze the pre-treated serum samples in the positive and negative ion mode according to the established conditions. Serum ESI-MS metabolic profile data was input into the Progenesis QI software for data preprocessing. After the peaks were matched, extracted and normalized, the ions were normalized and unsupervised PCA was performed on all ions. Then, supervised OPLS-DA is performed to calculate the contribution value of each ion reflecting the clustering and inter-group separation in the group, and *T*-testing the normalized data. Statistically significant ions with *P* < 0.05 difference between groups were selected as candidate ions and element matching and secondary identification were performed.

#### Biomarkers Identification and Metabolic Pathway

Firstly, the Rt-m/z of the important metabolite ions is locked, and the ions are extracted in the TIC total ion chromatogram to extract the Mass, and then the elemental composition analysis is performed to determine the possible chemical formula. After that searched on the Human Metabolome Database (HMDB) and Metaboanalyst website, and initially matched the mass spectrometry information to estimate the possible attribution of the metabolites. Based on the massfragment software attached to the Masslynx software system, the obtained compounds and the secondary mass spectrum were used as prerequisites. The effectiveness of the metabolites was demonstrated by pyrolysis of the mass spectrometry and the possibility of chemical structure cleavage. MetPA (Metabolomics Pathway Analysis) is a specialized network tool for analyzing metabolic pathways with biological background in visual metabolomics data. MetPA combines a variety of advanced path analysis programs to analyze the topological characteristics of metabolite metabolic pathways to help identify a metabolic pathway most relevant to metabolomics research. Using this software, determine the serum metabolic pathway associated with the resulting biomarker.

#### Statistical Analysis

The data were treated with SPSS software, and the statistical results were represented by (x ± s). Univariate anova was used for inter-group comparison, and Bonferroni test was used for multiple comparison, *P* < 0.05 was considered to be statistically significant. *P* < 0.01 was considered to have an extremely significant statistical difference.

## Results

### Bone Mineral Density and Bone Biomechanics

**Figure 1:**
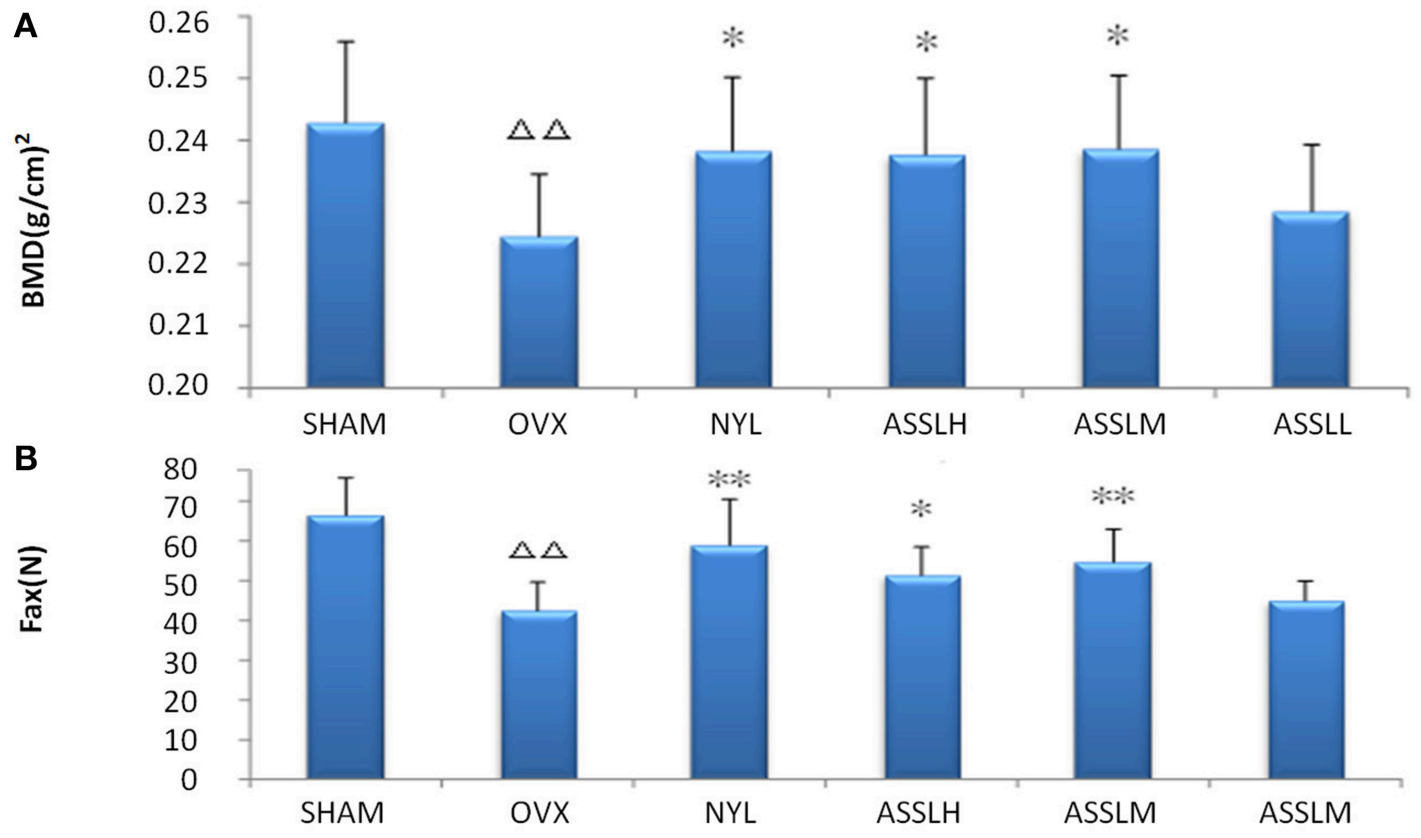
Effects of nylestriol and total lignans on the bone mineral density and the maximum load of ovariectomized osteoporosis rat model (compared with sham group, ^Δ^Δ*P* < 0.01; the OVX model group, ^*^P < 0.05, ^**^P < 0.01) **(A)** bone mineral density **(B)** maximum load.

Place the lumbar vertebrae (LV1-4) under the probe of the bone densitometer (DPX-MD, America), scan it with small animal software (Figure 1A), and place the lumbar vertebrae (LV5) at the universal material testing machine (Zwick/Roell, Germany) the maximum load of the concave experiment was measured (Figure 1B). The results displayed that the index of the OVX group decreased compared with the SHAM group (*P* < 0.01). Compared with the OVX group, NYL and ASSLH, ASSLM group were increased, which was statistically significant (*P* < 0.01, *P* < 0.05). The increase in the ASSLL group was lowly and it had no significant differences. Compared with the NYL, ASSLH, and ASSLM groups, the increase in bone density was similar, indicating that all three groups can improve bone mineral density reduction caused by ovariectomy, improve bone load capacity, and increase bone biomechanical properties.

### Bone Metabolism and Uterus Index Determination

**Figure 2:**
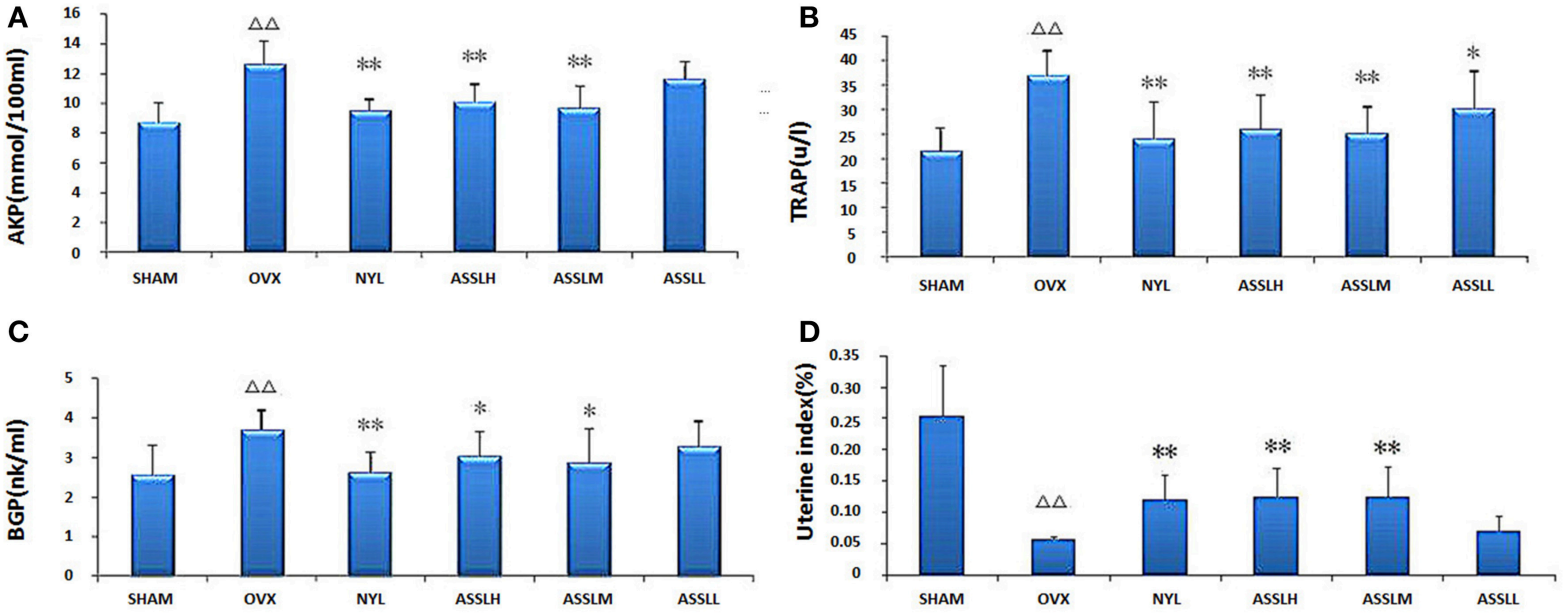
Effects of nylestriol and total lignans on the serum alkaline phosphatase, tartrate-resistant acid phosphatase and osteocalcin of ovariectomized osteoporosis rat model (compared with sham operation group, ^ΔΔ^*P* < 0.01; the OVX model group, ^*^P < 0.05, ^**^P < 0.01). **(A)** Alkaline phosphatase; **(B)** Tartrate-resistant acid phosphatase; **(C)** Osteocalcin; **(D)** Rat uterus index.

Serum alkaline phosphatase (AKP), serum tartrate-resistant acid phosphatase (TRAP) and serum osteocalcin (BGP) in bone metabolism were selected to evaluate the effect of ASSL on bone metabolism in ovariectomized rats. The results showed that compared with the SHAM, the AKP (Figure 2A), TRAP (Figure 2B), and BGP (Figure 2C) model groups were all increased (*P* < 0.01); compared with the OVX, NYL, ASSLH, ASSLM group can reduce the content of AKP, TRAP, BGP (*P* < 0.01, *P* < 0.05), the ASSLL group has a small reduction, no statistical significance; NYL, ASSLH, ASSLM group can significantly reduce ovariectomy. The increase of bone metabolism index caused by surgery indicates that ASSL can effectively inhibit bone over-transformation, regulate bone cell balance, and inhibit osteoblast cell disruption. Therefore, it can increase bone formation and decrease bone resorption, thus reducing bone turnover and regeneration rate.

The uterus index was measured by extracting the uterus of the rat. The results showed (Figure 2D): compared with the SHAM group, the uterus index of the OVX group decreased (*P* < 0.01); compared with the OVX group, the uterus index of the NYL, ASSLH, and ASSLM groups increased. The ASSLL group had a small increase and was not statistically significant. An increase in the uterus index indicates an increase in estrogen levels, but the three did not restore the uterus index to its original level.

### Metabolic Profiling

**Figure 3:**
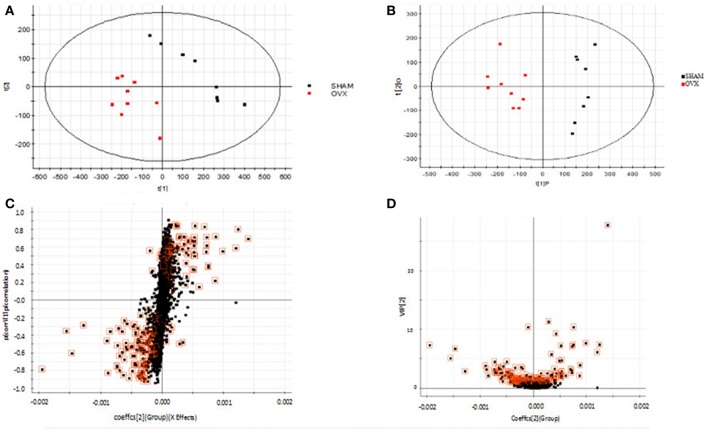
Metabolomics profiling of ovariectomized osteoporosis in positive ion mode at 7 weeks: **(A)** score plot of serum profile of sham operation group and model group scanned by PCA analysis. **(B)** Score plot of serum profile of sham operation group and model group scanned by OPLS-DA analysis. **(C)** S-plot of serum profile of sham operation group and model group scanned by OPLS-DA analysis. **(D)** VIP-plot of serum profile of sham operation group and model group scanned by OPLS-DA analysis.

**Figure 4:**
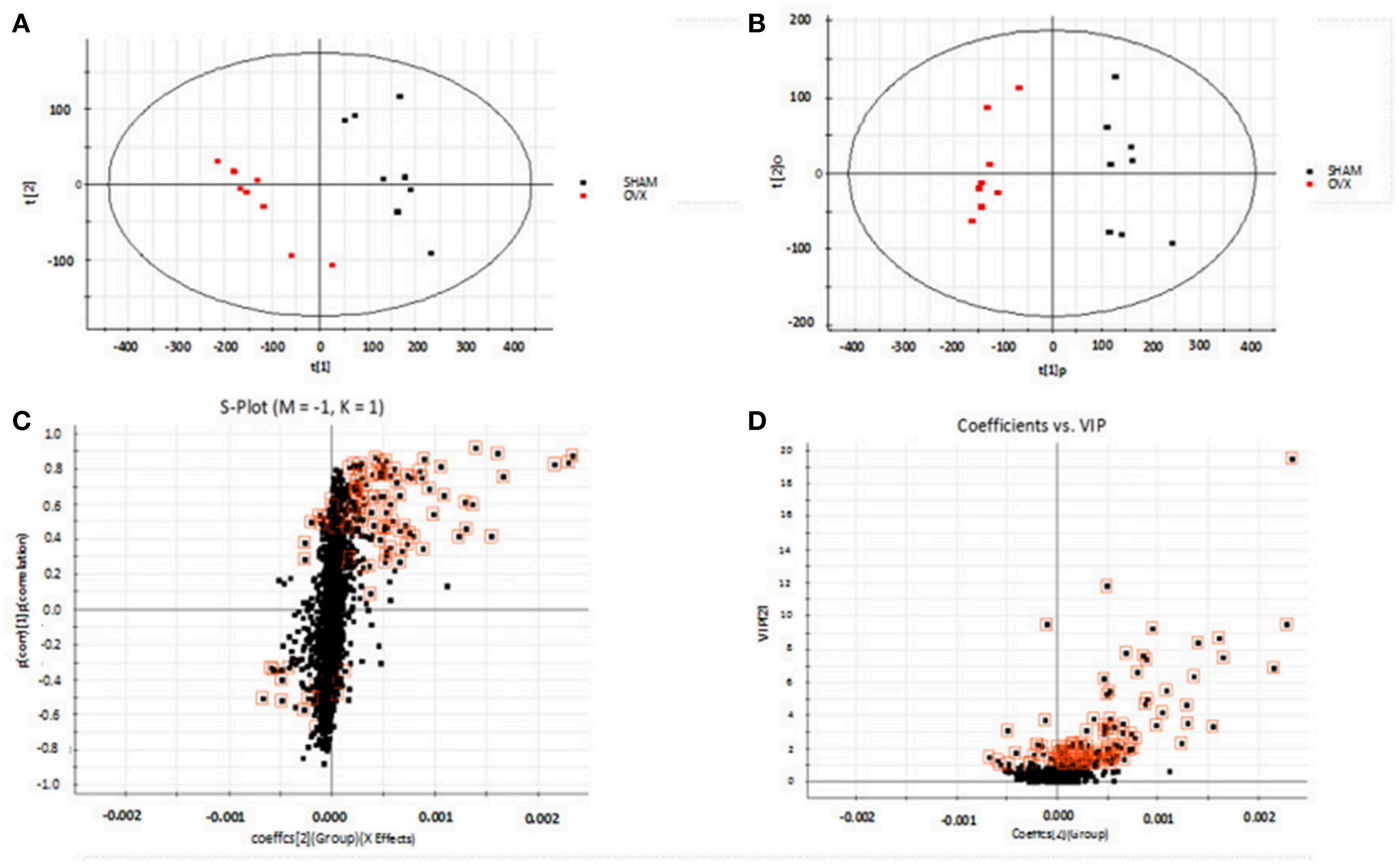
Metabolomics profiling of ovariectomized osteoporosis in negative ion mode at 7 weeks: **(A)** score plot of serum profile of sham operation group and model group scanned by PCA analysis. **(B)** Score plot of serum profile of sham operation group and model group scanned by OPLS-DA analysis. **(C)** S-plot of serum profile of sham operation group and model group scanned by OPLS-DA analysis. **(D)** VIP-plot of serum profile of sham operation group and model group scanned by OPLS-DA analysis.

**Figure 5:**
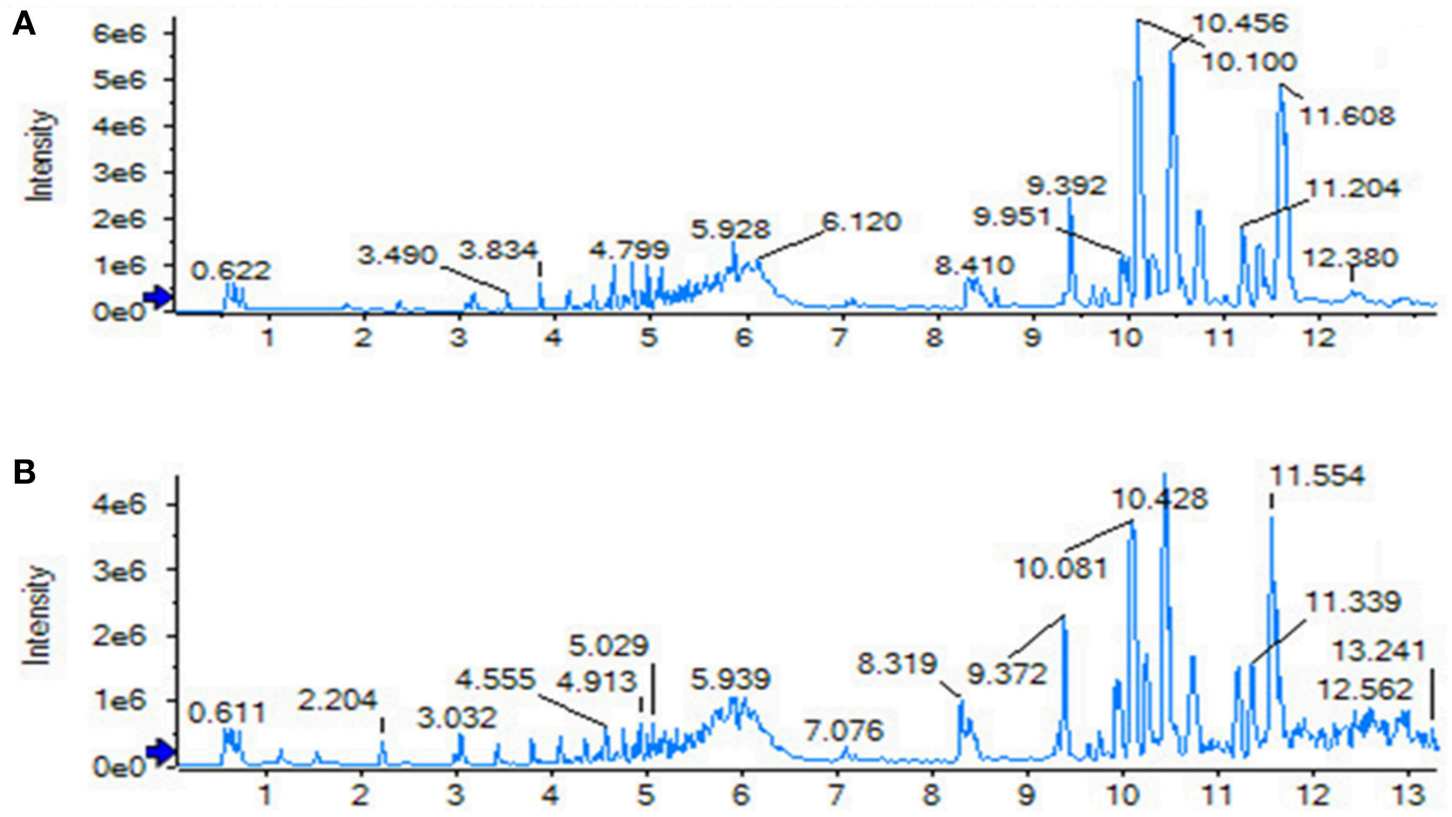
Serum TIC map in positive ion mode. **(A)** Sham operation group; **(B)** model group.

**Figure 6:**
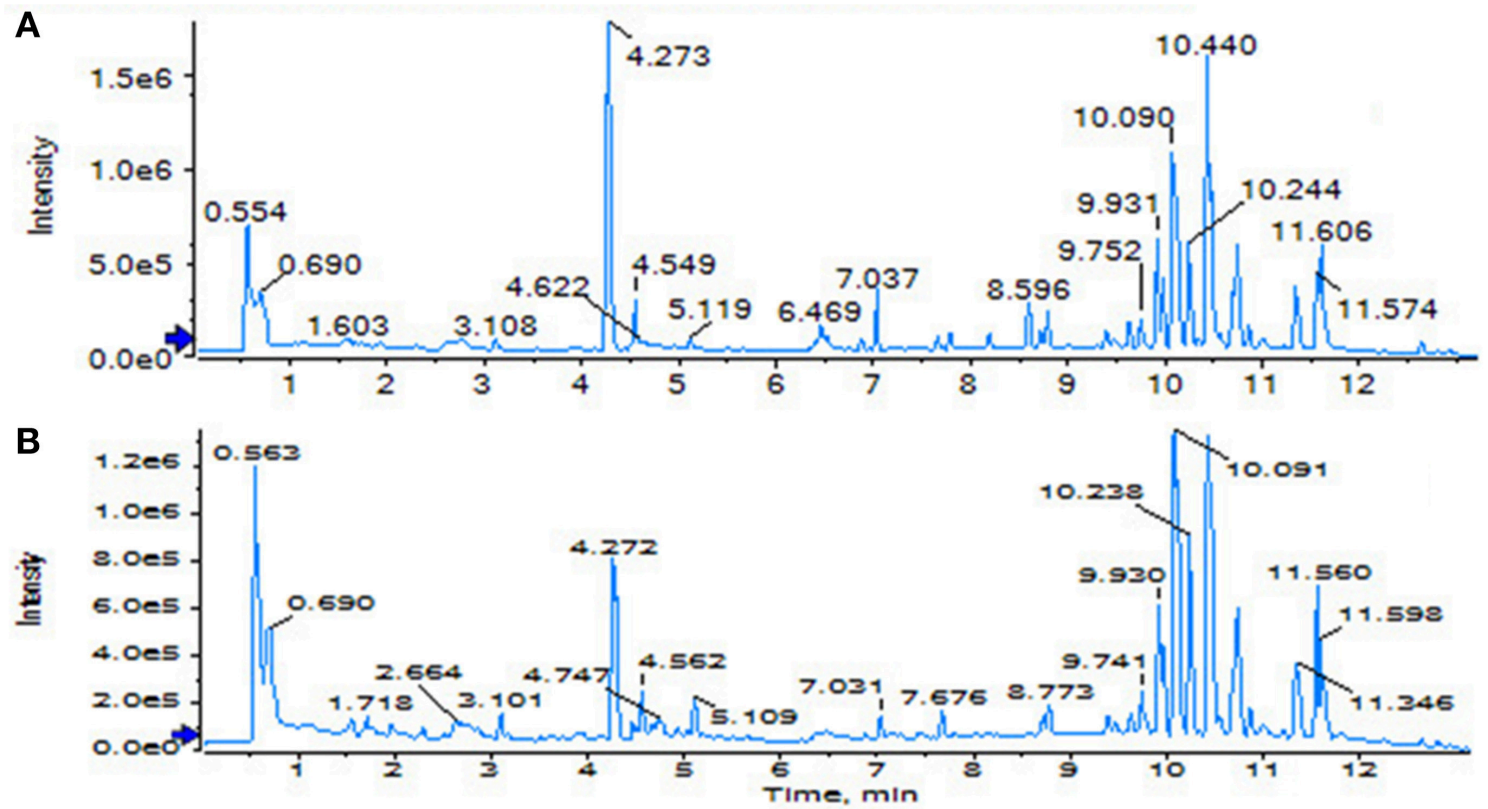
Serum TIC map in negative ion mode. **(A)** Sham operation group; **(B)** model group.

Using the above UPLC-MS conditions, the serum samples were processed in accordance with the serum sample processing method, and the serum samples were subjected to full-scan analysis in positive and negative ion mode. The serum ESI-MS metabolic profile data were input into the Progenesis QI software. After the peaks were matched, extracted and normalized, the ions were normalized, and all ions were analyzed in PCA to obtain the change between the groups. The trend of the score plot, positive (Figure 3A) negative (Figure 4A), showed that the osteoporosis model group and the sham operation group clustered significantly; indicating that the endogenous serum metabolic network of rats after membrane formation changed significantly. To find the endogenous metabolites that play a considerable role in the metabolism clustering separation, the above-mentioned serum sample metabolic profile was analyzed by OPLS-DA to obtain Score plot of positive mode (Figure 3B) and negative mode (Figure 4B). The osteoporosis rat model group was significantly separated from the blank control group, and the S-plot diagram was further positive (Figure 3C), negative mode (Figure 4C), the farther away from the far point in the figure, the greater the contribution rate. Further select ions with VIP value >1 in the VIP scatter plot as showed in the positive mode (Figure 3D) and negative mode (Figure 4D), and then combined with the VIP value >1 and the inter-group *T*-test results, ions with a *P*-value of < 0.05 were selected for structural identification as potential biomarkers. The metabolic profile fingerprint of the serum sample is shown in Figures 5, 6.

### Metabolite Identification

**Figure 7:**
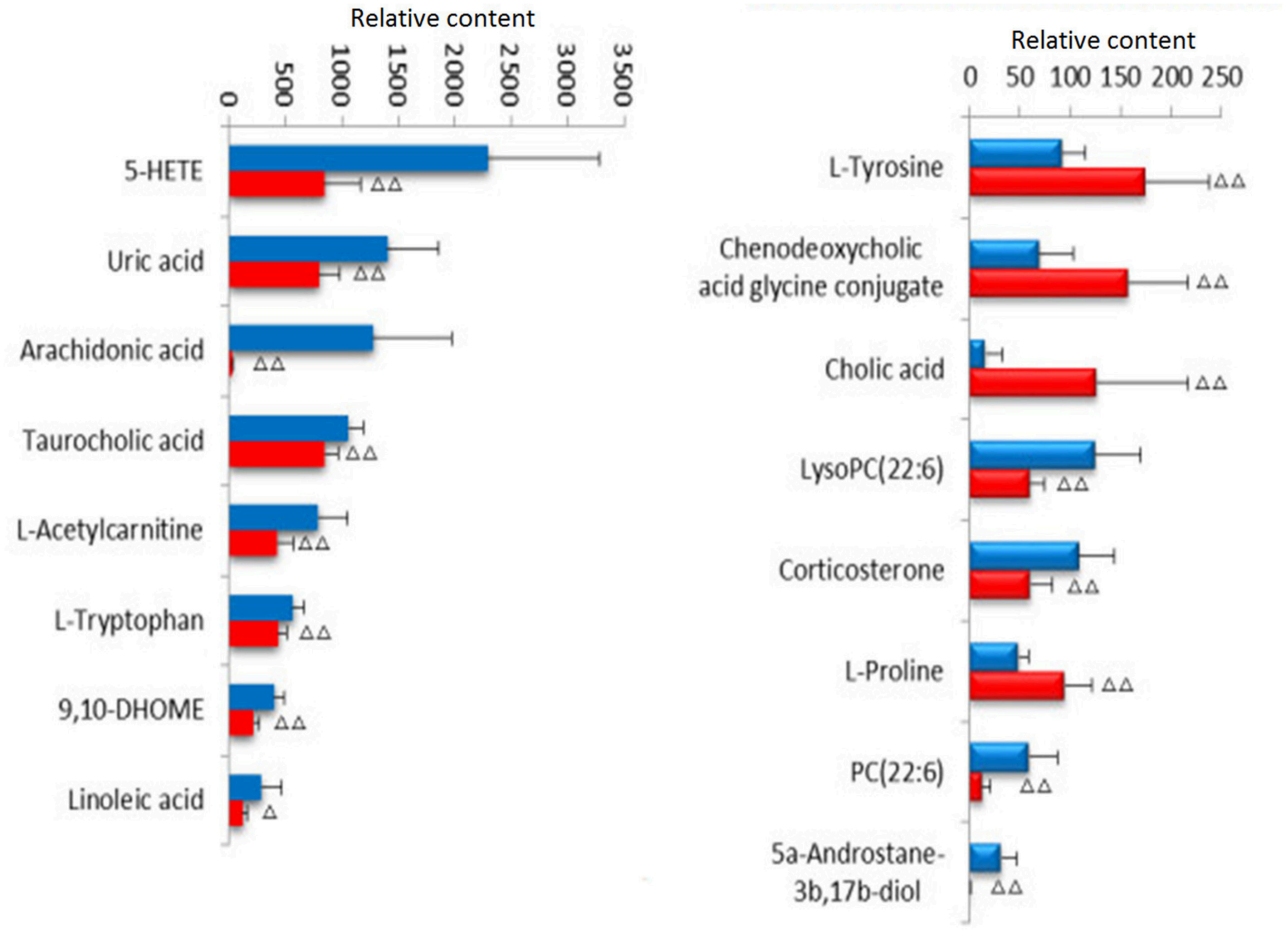
Changes of potential biomarkers in ovariectomized osteoporosis model. 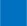, Sham operation group; 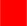, OVX Model group. Compare with sham operation, ^Δ^*P* < 0.05, ^ΔΔ^*P* < 0.01.

Lock the Rt_m/z of the important metabolite ions, and obtain the ions in the TIC total ion chromatogram to extract the Mass, and perform element composition analysis to determine the possible chemical formula. Based on the possible chemical formulas, accurate masses and secondary mass spectrometry data (MS/MS) of ions, the Human Metabolome Database (HMDB) and Metaboanalyst sites were searched to match the mass spectrometry information, and the possible attribution of metabolites was speculated. The effectiveness of the metabolites was demonstrated using the massfragment software included with the masslynx software system. In this study preliminarily identified 16 key metabolites, as showed in Table S2. The results of the secondary ion fragmentation information matching of these 16 compounds are shown in Table S3. The content of these potential biomarkers in the 12th week model group and the 12th week blank group were statistically analyzed (Figure 7).

### Biological Pathway and Functional Analysis

**Figure 8:**
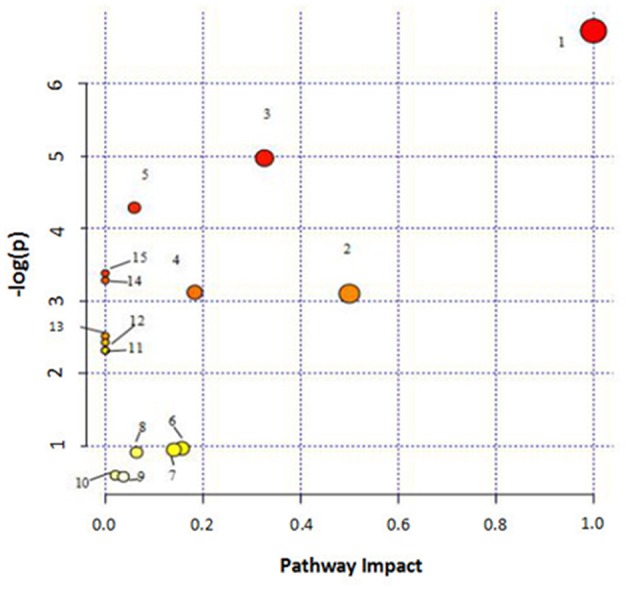
Main metabolic pathways of potential biomarkers. Linoleic acid metabolism; (2) Phenylalanine, tyrosine and tryptophan biosynthesis; (3) Arachidonic acid metabolism; (4) Glycerophospholipid metabolism; (5) Primary bile acid biosynthesis; (6) Tryptophan metabolism; (7) Tyrosine metabolism; (8) Arginine and proline metabolism; (9) Steroid hormone biosynthesis; (01) Purine metabolism; (11) alpha-Linolenic acid metabolism; (12) Taurine and hypotaurine metabolism; (13) Biosynthesis of unsaturated fatty acids; (14) Aminoacyl-t RNA biosynthesis; (15) Ubiquinone and other terpenoid-quinone biosynthesis.

MetPA (Metabolomics Pathway Analysis) is a specialized network tool for analyzing metabolic pathways with biological background in visual metabolomics data. MetPA combines many advanced path analysis programs to analyze the topological characteristics of metabolic pathways of metabolites can helps to determine one metabolic pathway most relevant to metabolomics research. MetPA analysis was performed on the identified 16 biopsy markers related to osteoporosis model, and 15 related metabolic pathways were obtained, including Aminoacyl-tRNA biosynthesis; ubiquinone and other terpenoid-quinone biosynthesis; phenylalanine, tyrosine and tryptophan biosynthesis; primary bile acid biosynthesis; taurine and hypotaurine taurine and hypotaurine metabolism; Arachidonic acid metabolism; glycerol phospholipid metabolism; biosynthesis of unsaturated fatty acids; tyrosine metabolism; fine Arginine and proline metabolism; purine metabolism; linoleic acid metabolism; tryptophan metabolism; steroid hormone biosynthesis, alpha-linolenic acid metabolism see Figure 8 for details. These results suggest that these endogenous metabolites strongly perturb the whole metabolic trajectory and are closely related to ovariectomized osteoporosis. Three closely related metabolic pathways were obtained, namely lipid metabolism, amino acid metabolism and nucleotide metabolism.

### Therapeutic Effects Analyses on Metabolite Profile

**Figure 9:**
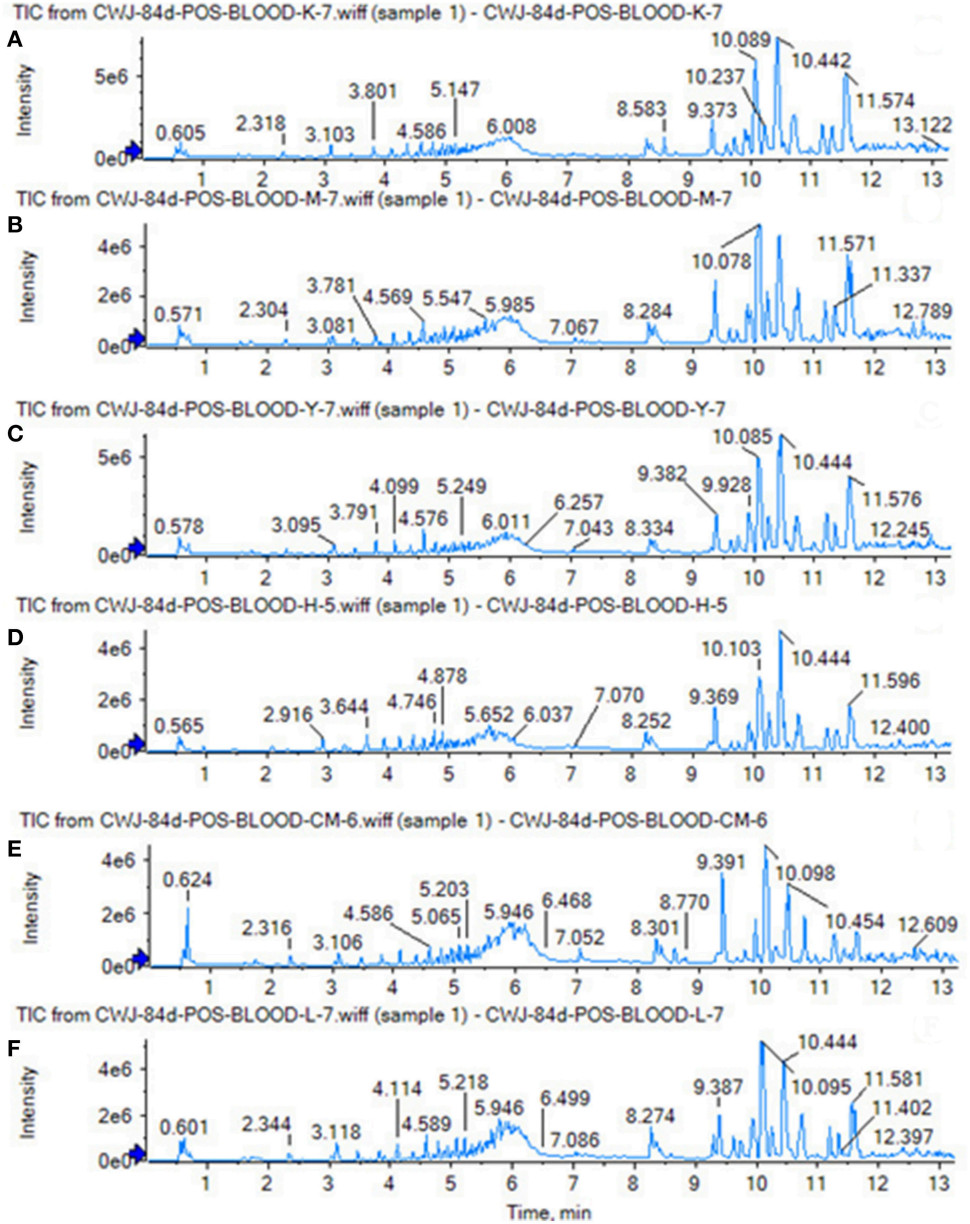
TIC of total lignans on the ovariectomized osteoporosis rat model in positive ion mode. **(A)** Sham operation group; **(B)** OVX Model group; **(C)** NYL; **(D)** ASSLH; **(E)** ASSLM; **(F)** ASSLL.

**Figure 10:**
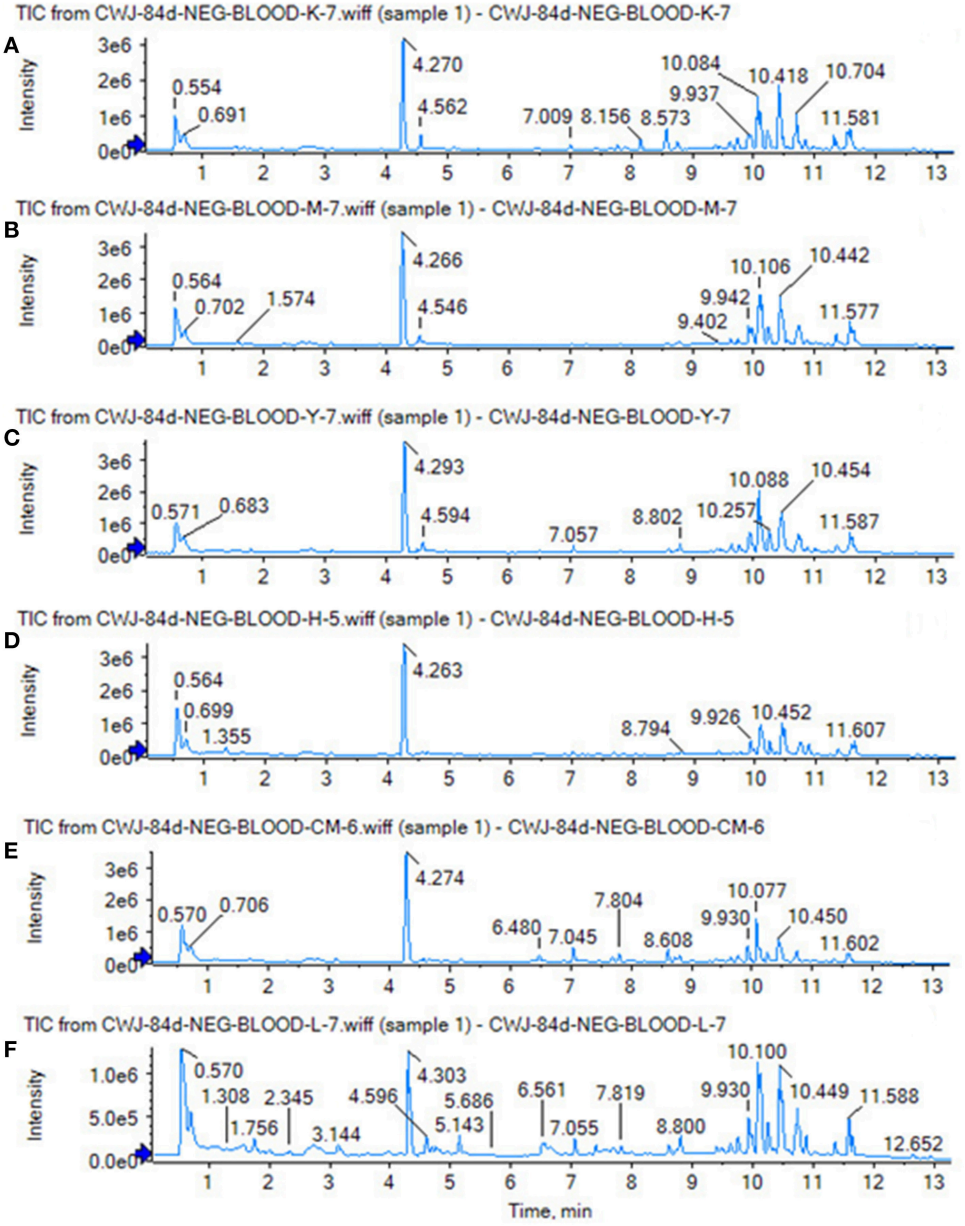
TIC of total lignans on the ovariectomized osteoporosis rat model in negative ion mode. **(A)** Sham operation group; **(B)** OVX Model group; **(C)** NYL; **(D)** ASSLH; **(E)** ASSLM; **(F)** ASSLL.

**Figure 11:**
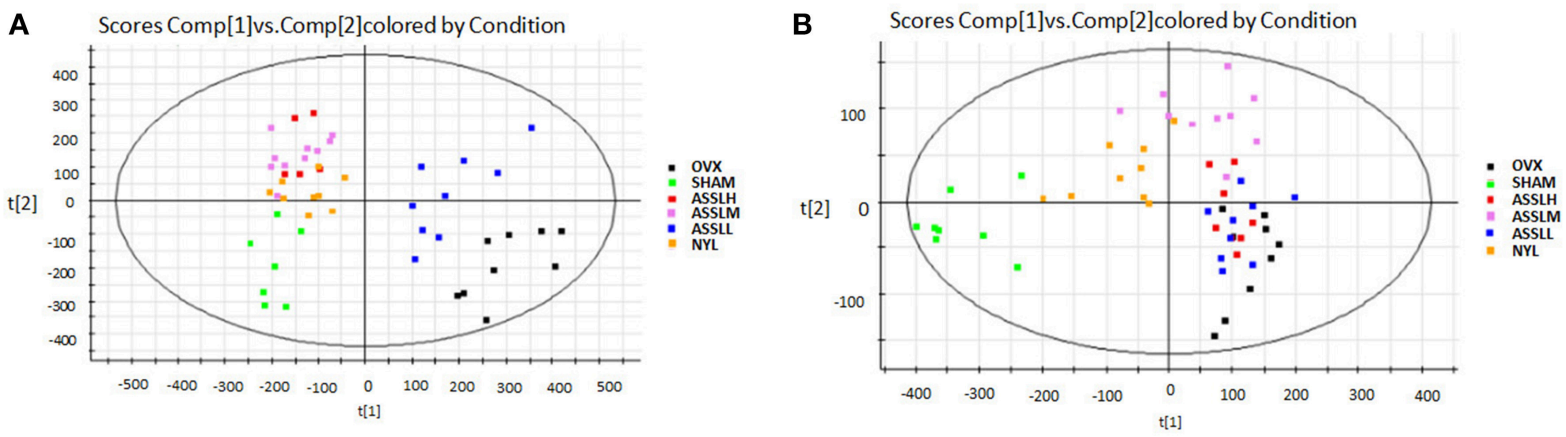
PCA scores plot of metabolic profile of total lignans on the ovariectomized osteoporosis rat model in both positive and negative mode. **(A)** Positive ion mode; **(B)** Negative ion mode.

The SHAM, ASSLH, ASSLM, ASSLL group of rats in the 12th week blood samples were processed according the foregoing method, positive and negative ions are completely scanned, and the metabolic profile of the three-dimensional information (retention time, mass-to-nucleus ratio, peak intensity) of each group is obtained as shown in Figures 9, 10. The blood metabolic profile data of the 12th week of each test group were imported into the QI software for data dimensionality reduction and mass spectrometry matrix information acquisition. Further, the EZinfo2.0 software module was used to perform unsupervised Principal Components Analysis (PCA) analysis on each group of data. Figure 11 shows the score plot showing the trend of changes between groups. It can be seen that the clusters in each group were clustered and separated between groups; The sham operated group was significantly separated from the ovariectomized osteoporosis model, indicating that the rat serum metabolism profile was significantly different from that of the sham operated rats after replication of the ovariectomized osteoporosis rat model; ASSLH and ASSLM intervention group clustering, and the vector position is closer to the SHAM group, which indicated that ASSL could delay the pathological process of ovariectomized osteoporosis model rats to some extent.

### Effects on Potential Biomarkers

**Figure 12:**
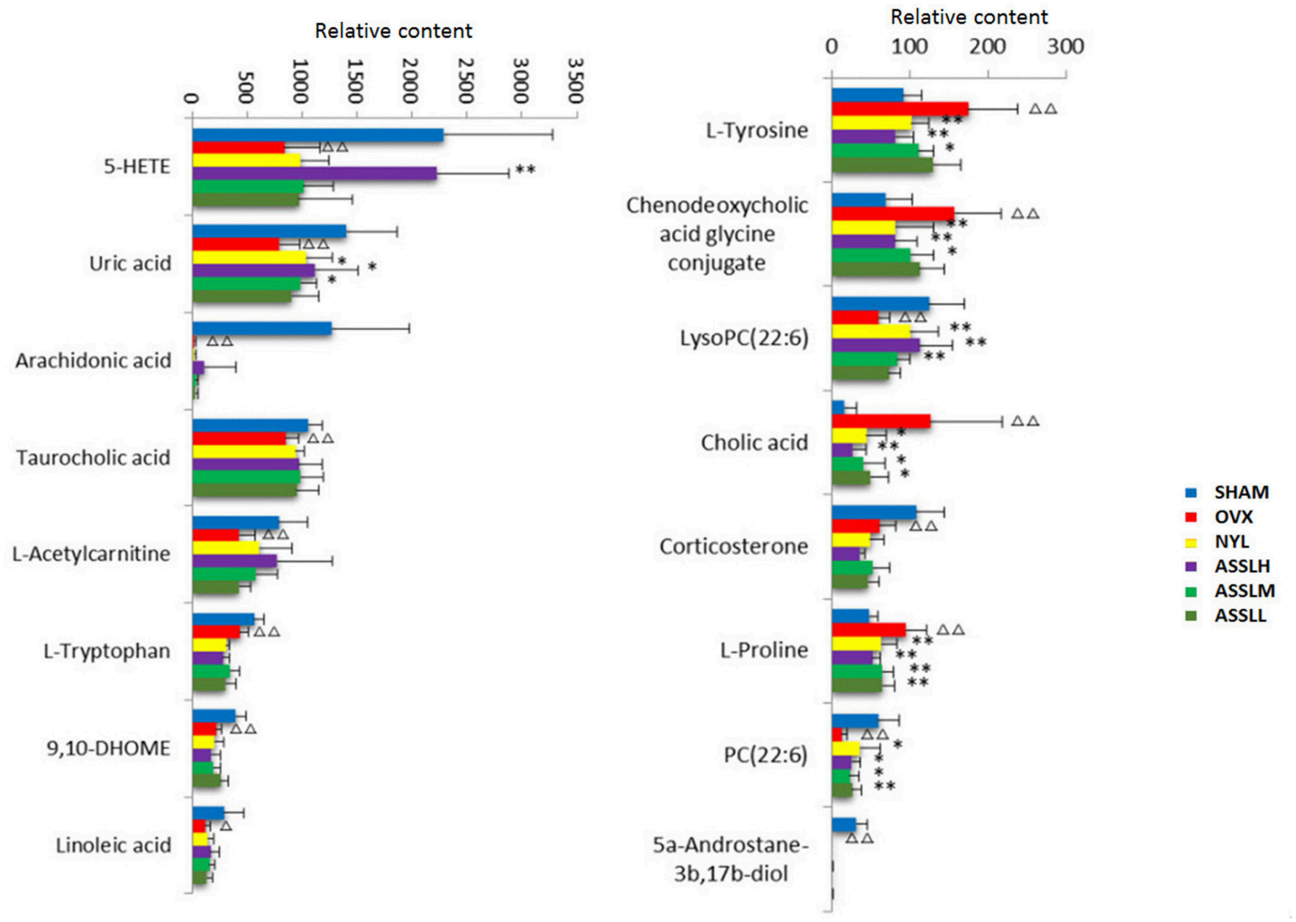
Changes of potential biomarkers in total lignans on the ovariectomized osteoporosis rat model. 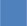, Sham operation group; 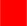, OVX Model group; 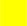, NYL; 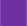, ASSLH; 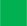, ASSLM; 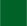, ASSLL (Compare with sham group, ^Δ^*P* < 0.05, ^ΔΔ^*P* < 0.01; compare with OVX Model group, ^*^P < 0.05, ^**^P < 0.01).

To analyze the changes of the potential biomarkers of osteoporosis model induced by ovariectomy in different doses of ASSL enrichment and oral administration of positive control drugs, It was found that the application of ASSL enrichment intervention can affect the potential biomarkers of osteoporosis model induced by ovariectomy, and its content is adjusted back to the direction of the sham operation group. Among the 16 potential biomarkers identified, nylestriol intervention could be adjusted back to 11 and 7 were statistically different; the ASSLH enrichment could be adjusted back to 11 and 8 were statistically different; the dose intervention of ASSLM enrichment could be adjusted back to 11 and 7 were statistically different. The ASSLL enrichment could be adjusted back to 10 and 3 were statistically different. There are 10 common markers that can be called back at three doses, respectively phospholipids (PC (22:6 (4Z, 7Z, 10Z, 13Z, 16Z, 19Z)/18:4 (6Z, 9Z, 12Z, 15Z))), L-proline, uric acid, L-tyrosine, taurocholic acid, chenodeoxycholic acid glycine conjugate, cholic acid, phospholipids (LysoPC (20:3 (5Z, 8Z, 11Z))), arachidonic acid (5-HETE), linoleic acid (Figure 12). Studies on the metabolic pathways of potential biomarkers showed that the main metabolic pathways were lipid metabolism, amino acid metabolism, and nucleotide metabolism. It is suggested that the ASSL exert its role in preventing and treating osteoporosis by interfering with the above metabolic pathways.

## Discussion

PMOP is a common disease associated with aging, mainly in postmenopausal women. Due to lack of estrogen, bone mass is reduced and bone tissue structure changes, bone fragility is increased, fracture is easy, and pain and bone deformation caused by fracture the occurrence of comorbidities and other serious effects on life of the elderly. Bone mineral density, bone biomechanics and bone metabolism indicators are still important indicators for evaluating postmenopausal osteoporosis. In this study, a classic postoperative model of osteoporosis was established by classical surgical castration. The study found that at 12 weeks after model replication, the bone density of LV1-4 in the model group decreased significantly compared with the SHAM group (*p* < 0.01), the maximum load of LV5 concave experiment was significantly decreased (*p* < 0.01), AKP, RTAP, BGP in rat serum increased significantly (*p* < 0.01), and the uterus index decreased significantly (*p* < 0.01). Changes in these indicators indicate that ovariectomy surgery replicates the postmenopausal osteoporosis model, which results in decreased bone density, decreased bone strength, increased bone resorption, increased metabolic bone formation, and uterine atrophy. In the rats treated with different doses ASSL, the bone mineral density and bone biomechanical properties of the rats in each administrative group increased, and the serum levels of AKP, RTAP, and BGP decreased, which significantly increased the uterus index. The role of the lignans group in ASSL was extremely significant.

**Figure 13:**
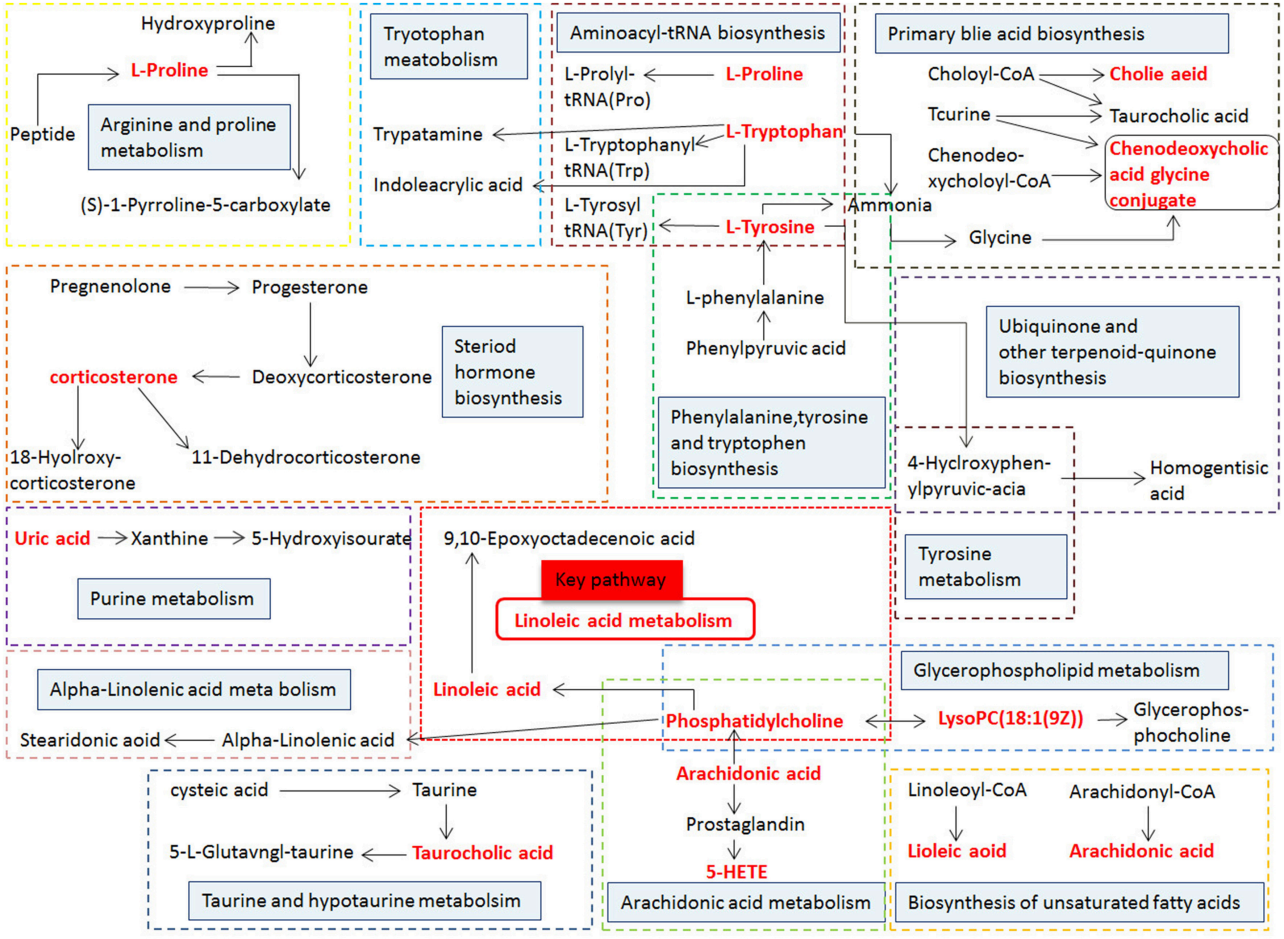
Correlation networks of the potential biomarkers based on the KEGG. The red font represents the biomarkers detected in this experiment and blue filled boxes represent related metabolic pathways.

Metabolomics analysis was used to analyze the serum of ovariectomized osteoporosis model rats and blank control group to study the difference of endogenous small molecule metabolic profiles. It can be seen that the 12th week after model replication. Model group and blank group Clustering grouping is obvious. By querying HMDB, MetPA, KEGG, and other databases, 16 potential biomarkers with significant influence on clustering group are locked. Related metabolic pathways involve lipid metabolism, amino acid metabolism, nucleic acid metabolism, etc., the roadmap of biomarkers and metabolic pathways is shown (Figure 13), suggesting that the above metabolic pathway may be a potential biochemical mechanism of PMOP. After oral administration of different doses of ASSL, it was preliminaries determined that 10 biomarkers that can be adjusted in the high, medium and low dose groups of ASSL were associated with key metabolites by tracking. The metabolic pathway changes, and the main metabolic pathways involved ASSL include lipid metabolism, amino acid metabolism, and nucleotide metabolism.

Unsaturated fatty acids are composed of monounsaturated fatty acids and polyunsaturated fatty acids, which are essential fatty acids for human body. Polyunsaturated fatty acids include arachidonic acid, linolenic acid, and linoleic acid. Among them, linoleic acid can be converted into arachidonic acid in the body, which is called n6 series. Related studies have shown that n6 series polyunsaturated fatty acids can reduce the incidence of fractures (Orchard et al., 2013). Because it has the effect of promoting the growth of bone cells and increasing bone mineral density, unsaturated fatty acid metabolism disorder can cause osteoporosis (Harris et al., 2015). In this research, The researchers observed that the content of linoleic acid in the blood of rats was decreased after ovariectomy in rats, and it was speculated that the biosynthesis of unsaturated fatty acids was destroyed, which may lead to osteoporosis.

Amino acid metabolism is a relatively important part of life activities which is the basic unit of macromolecular protein. It is indispensable for normal metabolism in the human body. Some amino acids can also be transformed in the body. Related literature has shown that amino acids are beneficial for the treatment of osteoporosis (Torricelli et al., 2003). Amino acids are synthetic raw materials for hemoglobin and leukocytes, which have a certain effect on the prevention of osteoporosis in middle-aged and elderly people. When amino acid metabolism is abnormal, it will accumulate in the body, affecting the synthesis of proteins and hindering the synthesis of proteins in bone. Tyrosine is an important amino acid in the body and can produce a variety of metabolites. It is a major raw material for the synthesis of thyroxine. In the tyrosine metabolic pathway of this experiment, L-tyrosine content in ovariectomized osteoporosis model rats was higher than that in the blank group. It is speculated that the tyrosine content in the model group is increased, so in the model group thyroid gland the level of the hormone may also be elevated, indicating that it is abnormal in the synthesis, storage, and release in the body, and the hormone levels and biochemical indicators in the hypothalamic-pituitary-thyroid axis are disordered. Related literature has shown that excessive thyroxine can cause osteoporosis, which is consistent with related literature reports (Leb et al., 1994). Proline in bone organic matter is a key component of collagen. Proline (Szabados and Savouré, 2010) can increase the activity of acid phosphatase (ACP) in blood and increase the activity of alkaline phosphatase (ALP) of the osteoporosis model. This indicates that the estrogen level of the rat ovary is reduced and the bone turnover is accelerated.

Uric acid, as the final product of purine metabolism, is the main indicator of purine metabolism. Blood uric acid is a reducing substance in the body, which participates in the redox reaction in the body, has strong antioxidant capacity, and can scavenge free radicals against DNA damage (Johnson et al., 2003; Sautin and Johnson, 2008; Chen et al., 2015). Epidemiological studies have reported the possible effects of uric acid on osteoporosis. Blood uric acid may have a dual effect on bone. It is generally considered that physiological concentration of blood uric acid has an anti-osteoporosis effect. On the one hand, uric acid has a certain protective effect on bone metabolism, which is believed to be related to its antioxidant function (Ahn et al., 2013; Beyazit and Pek, 2018). Uric acid is also positively correlated with the level of parathyroid hormone (PTH) (Chikura et al., 2009; Hui et al., 2012) and can also affect bone metabolism by regulating the activity of 1α-hydroxylase. On the other hand, oxidative stress reaction can lead to bone loss and participate in the mechanism of osteoporosis (Wauquier et al., 2009), which may be the crystallization deposition of uric acid, which reduces the activity of alpha-hydroxylase in the kidney and reduces the ability of the intestinal tract to absorb calcium.

Metabolomics is a newly developed discipline that mainly studies small molecular metabolites (MW < 1000) as substrates and products of various metabolic pathways, it is a branch of system biology based on group index analysis, high-throughput detection and data processing, and aiming at information modeling and system integration (Wu et al., 2018). Metabolomics plays an important role in exploring metabolic disorder metabolism-related diseases (Zhang et al., 2013b, 2014b,c,d, 2018a; Wang et al., 2014; Liu et al., 2018; Sun et al., 2018), biomarkers identification (Wang et al., 2015a; Zhang et al., 2015b,c; Zhao et al., 2016; Song et al., 2017; Ren et al., 2018) and response to treatment (Wu et al., 2011; Dong et al., 2012; Sun et al., 2013; Wang et al., 2013a, 2016; Zhang et al., 2013a, 2018b). In this study, the metabolomics study was used to analyze serum metabolism of the ovariectomized bone rat model and to observe the changes of ASSL during the treatment. Sixteen potential differential metabolic markers were found to be associated with postmenopausal osteoporosis, refer to 15 metabolic pathways. After treatment with ASSL, 10 biomarkers can be significantly adjusted (Table S4) to regulate the biosynthesis of unsaturated fatty acids, linoleic acid metabolism, arachidonic acid metabolism, arachidonic acid metabolism, arachidonic acid metabolism, tyrosine metabolism, purine metabolism, glycerol phosphoric acid metabolism, etc., and the metabolic expression is in the direction of delaying PMOP. For the first time, the prevention and treatment of oral ASSL on PMOP were described at the level of metabolic pathways, and the effectiveness and time potential of metabolomics methods for the study of traditional Chinese medicine interventions was demonstrated.

## Conclusion

In this study, the high-throughput metabolomics method was used to evaluate the efficacy of total lignans from ASS against ovariectomized osteoporosis rat, and 16 potential biomarkers were finally identified and involved in 15 related metabolic pathways. ASSL could regulate 10 biomarkers of them and mainly adjusts metabolic pathways include unsaturated fatty acid biosynthesis, linoleic acid metabolism, and arachidonic acid metabolism, primary bile acid synthesis, tyrosine metabolism, etc. It showed that the ASSL could affect the endogenous metabolites related metabolic mechanism, offers a pharmacological basis of the ASSL for PMOP treatment, and provide evidences for the development of new drugs for PMOP.

## Ethics Statement

The experimental procedures were approved by the Animal Care and Ethics Committee at Heilongjiang University of Chinese Medicine and all experiments were performed in accordance to the declaration of Helsinki.

## Author Contributions

XW conceived and designed the experiments. AZ, ZM, HS, YZ, and JL performed the experiment. ZM, HS, YZ, and JL analyzed the data. AZ guided the experiment. ZM wrote the paper. AZ revised the paper. All authors read and approved the final manuscript.

### Conflict of Interest Statement

The authors declare that the research was conducted in the absence of any commercial or financial relationships that could be construed as a potential conflict of interest.
