# Supplementary material for: High-Throughput Metabolomics Evaluate the Efficacy of Total Lignans From Acanthophanax Senticosus Stem Against Ovariectomized Osteoporosis Rat
Source: Front Pharmacol. 2019 May 29;10:553. doi: 10.3389/fphar.2019.00553 (PMC6548904; doi:10.3389/fphar.2019.00553)
Supplement: Supplementary file 1 [file Data_Sheet_1.doc]

**
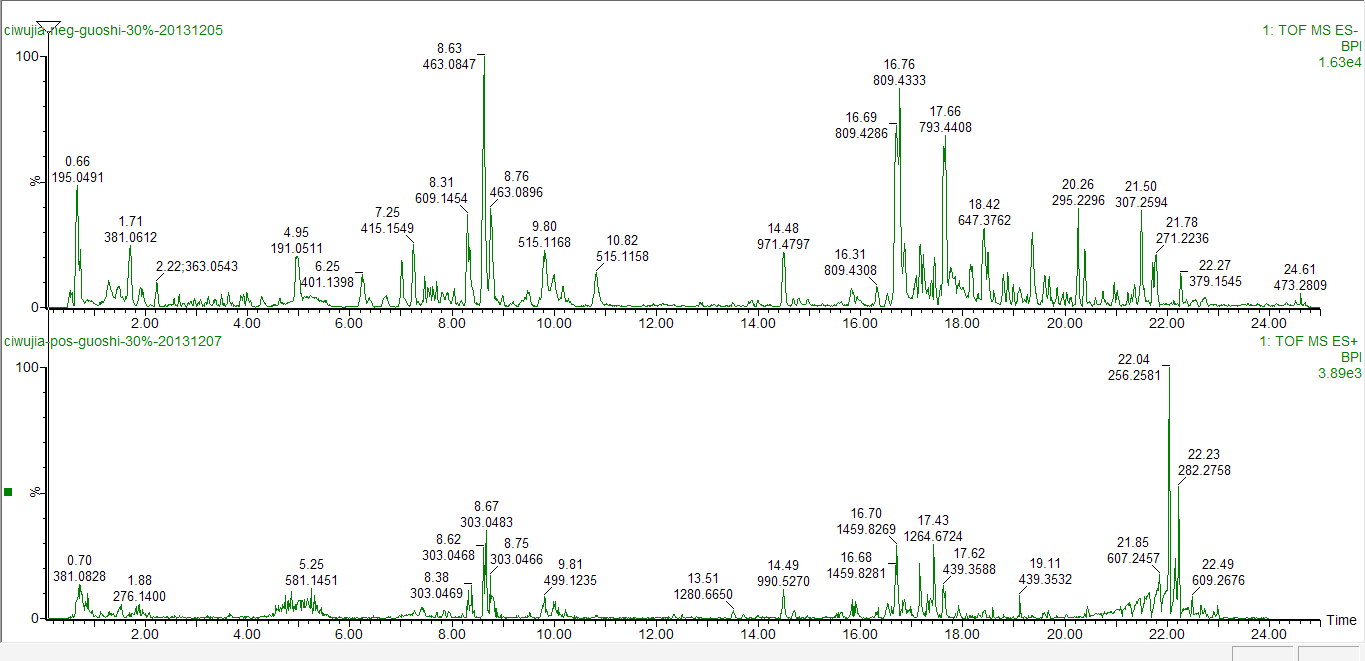
**

Fig S1. Identification and structural characterisation of compounds detected by UPLC-HDMS.

**Table S1** Identification and structural characterization of compounds detected in the *Acanthophanax senticosus* stem.

| NO. | Rt  (min) | [M-H]^-^(m/z) | Molecular formula | Error (ppm) | Fragment ions (m/z) | Compounds identified |  |
| --- | --- | --- | --- | --- | --- | --- | --- |
| 1 | 0.703 | 367.0513 | C12H16O13 | -4.6 | 191[M-H-C_6_H_8_O_6_] 129[M-H-C_7_H_10_O_9_] 113.[M-H C_7_H_10_O_10_] 85[M-H-C_9_H_14_O_10_] | 4-O -Galactopyranuronosyl- galactopyranuronate | 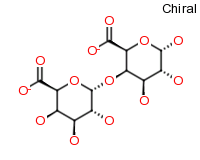 |
| 2 | 0.902 | 341.1084 | C12H22O11 | -2.6 | 179[M-H-C_6_H_10_O_5_] 161[M-H-C_6_H_12_O_6_] 143[M-H-C_6_H_14_O_7_] 113[M-H-C_7_H_16_O_8_] | Sucrose | 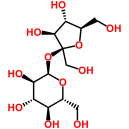 |
| 3. | 0.756 | 357.1033 | C12H22O12 | -1.1 | 195[M-H-C_6_H_10_O_5_] 165.[M-H-C_6_H_8_O_7_]161[M-H- C_6_H_12_O_7_] 129[M-H-C_7_H_16_O_8_] 75[M-H-C_10_H_18_O_9_] | 4-(b-D-Galactosido)-D-gluconic Acid | 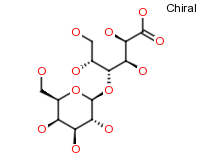 |
| 4 | 1.124 | 503.1612 | C18H32O16 | 0.8 | 221[M-H-C_10_H_18_O_9_] 131[M-H-C_13_H_24_O_12_]113[M-H-C_13_H_26_O_13_]101[M-H-C_14_H_26_O_13_] | Melezitose | 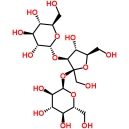 |
| 5 | 1.534 | 191.0192 | C6H8O7 | -5.2 | 173[M-H-H_2_O] 147[M-H-CO_2_] 129[M-H-CH_2_O_3_] 111[M-H- CH_4_O_4_] 87[M-H-C_3_H_4_O_4_] 67[M-H-C_3_H_8_O_5_] | 5-dehydro-4-deoxy-D-glucaric acid | 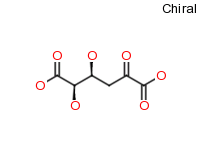 |
| 6 | 2.606 | 383.1553 | C15H28O11 | +1.0 | 251[M-H-C_5_H_8_O_4_] 161[M-H-C_9_H_18_O_6_] 113[M-H-C_10_H_22_O_8_] 101[M-H- C_10_H_18_O_9_]  85[M-H-C_10_H_18_O_10_]  71[M-H-C_11_H_20_O_10_] | 3-Hydroxy-2-butanyl6-O-[3,4-dihydroxy-4-(hydroxymethyl)tetrahydro-2-furanyl] -glucopyranoside | 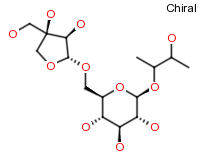 |
| 7 | 2.564 | 315.0716 | C13H16O9 | -1.9 | 153[M-H-C_6_H_10_O_5_] 109[M-H-C_7_H_10_O_7_] | Protocatechuic acid-glucoside | 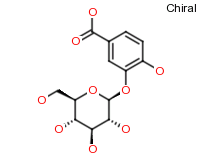 |
| 8 | 2.649 | 371.097 | C_16_H_20_O_10_ | -3.0 | 353[M-H-H_2_O]^-^191[M-H-C_9_H_8_O_4_]^-^179[M-H-C_7_H_12_O_6_]^-^173[M-H-C_9_H_10_O_5_]^-^ 161[M-H-C_7_H_14_O_7_]^-^ 155[M-H-C_9_H_12_O_6_]^-^135[M-H-C_8_H_12_O_8_]^-^137[M-H-C_9_H_14_O_7_]^-^ | (1S,3R,4R,5R)-3-{[(2E)-3-(3,4-Dihydroxyphenyl)-2-propenoyl]oxy}-1,4,5-trihydroxycyclohexanecarboxylic acid hydrate (1:1) | 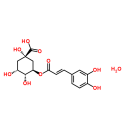 |
| 9 | 2.649 | 461.1295 | C19H26O13 | +3.0 | 329[M-H-C5H6O4] 311[[M-H- C5H10O5] 167[M-H-C11H18O9] 109[M-H-C13H20O11] | Methyl 5-({3,4-dihydroxy-4-(hydroxymethyl)tetrahydro-2-furanyl] -glucopyranosyl}oxy)-2-hydroxybenzoate | 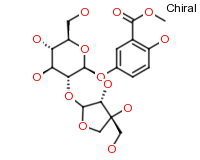 |
| 10 | 2.848 | 677．1929 | C_28_H_38_O_19_ | -3.7 | 515[M-H-C_6_H_10_O_5_]^-^353[M-H-C_12_H_20_O_10_]^-^191[M-H-C_21_H_26_O_13_]^-^179[M-H-C_19_H_30_O_15_]^-^  173[M-H-C_21_H_28_O_14_]^-^155[M-H-C_21_H_30_O_15_]^-^161[M-H-C_19_H_32_O_16_]^-^135[M-H-C_20_H_30_O_17_]^-^ | caffeoylquinic acid diglucoside |  |
| 11 | 2.870 | 353.1448 | C14H26O10 | -2.3 | 221[M-H-C_5_H_8_O_4_] 161[M-H- C_8_H_16_O_5_] 147[M-H- C_9_H_18_O_5_] 113[M-H- C_9_H_20_O_7_] 101[M-H- C_10_H_20_O_7_] 85[M-H- C_10_H_20_O_8_] 71[M-H-C_11_H_22_O_8_] | isopropyl -apiofuranosyl-(1->6)- glucopyranoside | 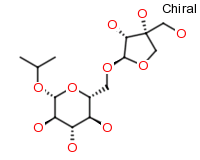 |
| 12 | 2.932 | 397.1710 | C16H30O11 | -1.0 | 353[M-H- CO_2_] 251[MH- C_6_H_10_O_4_] 221[M-H- C_6_H_8_O_6_] 161[M-H- C_10_H_20_O_6_] 113[M-H- C_10_H_20_O_9_] 101[M-H- C_11_H_20_O_9_] 85[M-H-C_12_H_24_O_9_] 71[M-H- C_12_H_22_O_10_] | Butyl 4/6-O-galactopyranosyl-β-D-glucopyranoside | 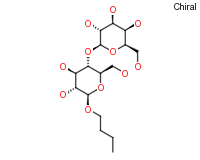 |
| 13 | 2.996 | 515.1401 | C22H28O14 | 0.4 | 353[M-H- C_6_H_10_O_5_]^-^191[M-H-C_15_H_16_O_8_]^-^ | caffeoylquinic acid glucoside |  |
| 14 | 3.038 | 315.0716 | C13H16O9 | -1.0 | 153[]M-H- C6H10O5] 109[MH- C7H10O7] | 3-Carboxy-4-hydroxy-phenoxy glucoside | 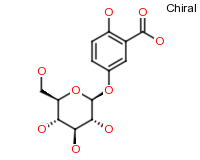 |
| 15 | 3.081 | 329.0873 | C14H18O9 | 0.3 | 167[M-H-C6H10O5] 152[M-H- C7H13O5] | 6-O-(4-Hydroxy-3-methoxybenzoyl)- glucopyranose | 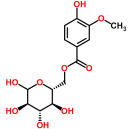 |
| 16 | 3.174 | 487.1088 | C21H28O13 | -0.4 | 337[M-H- C5H10O5] 247[M-H- C10H8O7] 192[M-H- C11H19O9] 164[M-H- C12H19O10] 137[M-H- C13H18O11] | [(2S,3R,4S,5S,6R)-3,4,5-trihydroxy-6-[[(2S,3R,4S,5R)-3,4,5-trihydroxytetrahydropyran-2-yl]oxymethyl]tetrahydropyran-2-yl] (Z)-3-(4-hydroxy-3-methoxy-phenyl)prop-2-enoate | 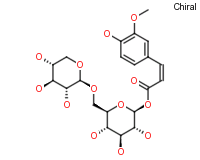 |
| 17 | 3.249 | 353.0873 | C16H18O9 | -2.5 | 191[M-H-C_6_H_10_O_5_] | Scopolin | 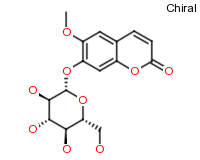 |
| 18 | 3.343 | 515.1401 | C22H28O14 | 3.3 | 353[M-H- C_6_H_10_O_5_]^-^191[M-H-C_15_H_16_O_8_]^-^ | caffeoylquinic acid glucoside |  |
| 19 | 3.321 | 153.0188 | C7H6O4 | -3.9 | 109[M-H- CO_2_]- | Protocatechuic acid | 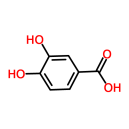 |
| 20 | 3.459 | 515.1401 | C22H28O14 | 3.3 | 353[M-H- C_6_H_10_O_5_]^-^191[M-H-C_15_H_16_O_8_]^-^ | caffeoylquinic acid glucoside |  |
| 21 | 3.521 | 447.1139 | C18H24O13 | -1.6 | 315[M-H- C_5_H_8_O_4_] 163[M-H- C_12_H_12_O_8_] 152[M-H-C_11_H_19_O_9_] 108[M-H- C_12_H_19_O_11_] | 1-O-p-hydroxybenzoyl-β-D-apiofuranosyl-(1--&gt;6)-β-D-glucopyranoside | 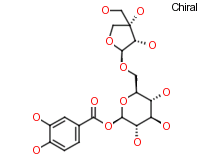 |
| 22 | 3.690 | 353.0873 | C16H18O9 | +0.3 | 191[M-H- C_9_H_6_O_3_] 179[M-H- C_7_H_10_O_5_] 135[M-H- C_8_H_10_O_7_] 85[M-H- C_13_H_16_O_6_] | Neochlorogenic acid | 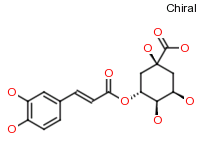 |
| 23 | 3.711 | 529.1557 | C23H30O14 | -2.1 | 367[M-H- C_6_H_10_O_5_]^-^193[M-H- C_13_H_20_O_10_]^-^ 173[M-H- C_16_H_20_O_9_]^-^ 155[M-H-C_16_H_22_O_10_]^-^ | Ferulyl quinic acid glucoside | 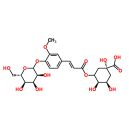 |
| 24 | 3.964 | 515.1401 | C22H28O14 | +1.0 | 353[M-H- C_6_H_10_O_5_]^-^191[M-H-C_15_H_16_O_8_]^-^ | caffeoylquinic acid glucoside |  |
| 25 | 4.079 | 443.1917 | C21H32O10 | -4.3 | 283[M-H- C6H8O5] 263[M-H- C6H12O6] 189[M-H- C10H22O7] 153[M-H- C12H18O8] 135[M-H- C16H20O6] 113[]M-H- C16H26O7] 101[M-H- C16H22O8] 71[M-H- C17H24O9] | 7-(Hydroxymethyl)-4-({[(2R,3R,4S,5S,6S)-3,4,5-trihydroxy-6-methoxytetrahydro-2H-pyran-2-yl]oxy}methyl)-1,4a,5,7a-tetrahydrocyclopenta[c]pyran-1-yl 3-methylbutanoate | 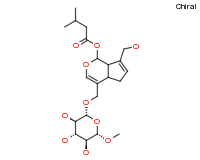 |
| 26 | 4.300 | 457.1710 | C21H30O11 | -1.1 | 277[M-H- C_6_H_12_O_6_] 251[M-H- C_7_H_10_O_7_] 233[M-H- C_8_H_16_O_7_]215[M-H- C_8_H_18_O_8_]189[M-H- C_10_H_20_O_8_] 171[M-H- C_13_H_18_O_7_] 123[M-H- C_15_H_26_O_8_] 113[M-H- C_15_H_20_O_9_] | 3,4-Dihydroxyallylbenzene 4-O-[rhamnopyranosyl-(1-->6)]- glucopyranoside | 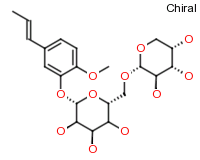 |
| 27 | 4.668 | 341.0873 | C15H18O9 | -2.9 | 181[M-H- C_6_H_8_O_5_] 179[M-H- C_6_H_10_O_5_] 135[M-H- C_7_H_10_O_7_] 109[M-H- C_9_H_12_O_7_]93[M-H- C_10_H_16_O_7_] | 1-Caffeoylglucose | 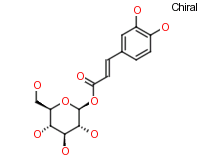 |
| 28 | 4.679 | 343.1029 | C15H20O9 | -4.7 | 181[M-H- C6H10O5] 137[M-H- C8H14O6] 121 [M-H-C11H10O5] 119[M-H- C10H8O6] 109[M-H- C9H14O7] 59[M-H- C13H16O7] | 4-Formyl-2,6-dimethoxyphenyl β-D-glucopyranoside | 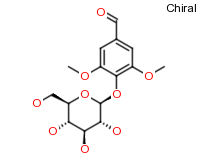 |
| 29 | 4.732 | 515.1401 | C22H28O14 | -3.9 | 353[M-H- C6H10O5]-191[M-H-C15H16O8]- | caffeoylquinic acid | 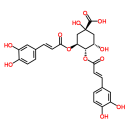 |
| 30 | 5.015 | 353.0814 | C16H18O9 | +4.8 | 191[M-H-C6H10O5] | Magnolioside | 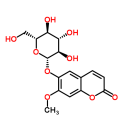 |
| 31 | 5.142 | 357.1186 | C16H22O9 | 0.0 | 195 [M-H-C6H10O5] | 10-Deoxygeniposidic acid | 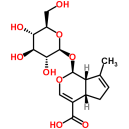 |
| 32 | 5.204 | 385.1135 | C17H22O10 | +5.2 | 223[M-H- C_6_H_10_O_5_]208[M-H- C_6_H_9_O_6_] 193[M-H- C_7_H_12_O_6_] 179[M-H- C_7_H_10_O_7_] 164[M-H- C_11_H_9_O_5_] 149[M-H- C_12_H_12_O_5_] | 1-O-sinapoyl-β-D-glucose /Methyl 1-(β-D-glucopyranosyloxy)-7-(hydroxymethyl)-1,7a-dihydrocyclopenta[c]pyran-4-carboxylate | 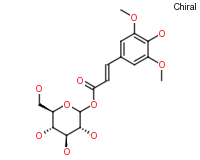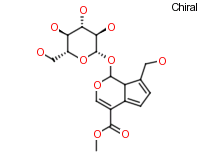 |
| 33 | 5.393 | 481.2285 | C21H38O12 | 2.3 | 326[M-H- C_10_H_19_O] 191[M-H- C_14_H_26_O_6_] 149[M-H- C_16_H_28_O_7_] 131[M-H- C_16_H_30_O_8_] 99[M-H- C_16_H_30_O_10_] | ((2R,3S,4S,5S)-3,4-DIHYDROXY-5-(HYDROXYMETHYL)-5-((2R,3S,4S,5S,6R)-3,4,5-TRIHYDROXY-6-METHOXY-TETRAHYDRO-2H-PYRAN-2-YLOXY)-TETRAHYDROFURAN-2-YL)METHYL NONANOATE | 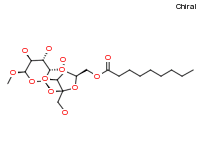  壬酸+2个糖 |
| 34 | 5.404 | 271.0970 | C16H16O4 | -1.5 | 256[M-H- CH_3_] 241[M-H- C_2_H_6_] | trans-4,4'-Dihydroxy-3,3'-dimethoxystilbene | 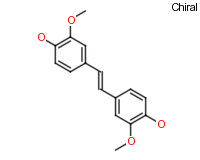 |
| 35 | 5.583 | 353.0873 | C16H18O9 | -4.5 | 191[M-H- C9H6O3] 179[M-H- C7H10O5] 135[M-H- C8H10O7] 85[M-H- C13H16O6] | Chlorogenic acid | 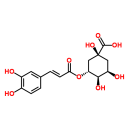 |
| 36 | 6.078 | 179.0344 | C9H8O4 | -1.1 | 135[M-H- CO2]- | Caffeic acid | 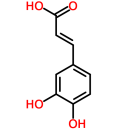 |
| 37 | 6.236 | 401.1448 | C18H26O10 | -2.5 | 269[M-H- C5H8O4] 161[M-H- C12H16O5] 125[M-H- C12H20O7] 113[]M-H- C13H20O7] 101[M-H- C13H16O8] 85[M-H- C13H16O9] 71[M-H- C15H22O8] | Phenyl 2-O-(6-deoxy-α-L-galactopyranosyl)-β-D-galactopyranoside | 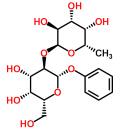 |
| 38 | 6.719 | 401.1448 | C18H26O10 | -4.0 | 269[M-H- C5H8O4] 161[M-H- C12H16O5] 125[M-H- C12H20O7] 113[]M-H- C13H20O7] 101[M-H- C13H16O8] 85[M-H- C13H16O9] 71[M-H- C15H22O8] | Benzyl 6-O-pentopyranosylhexopyranoside | 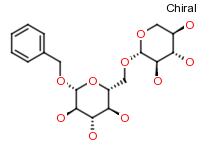 |
| 39 | 7.046 | 371.1283 | C17H24O9 | +5.1 | 327[M-H- CO2] 191[M-H- C6H12O6] 147[M-H- C11H12O5] 113[M-H- C12H18O6] 101[M-H- C12H14O7] 85[M-H- C14H22O6] | Syringin | 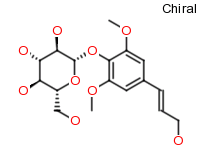 |
| 40 | 7.097 | 431.1917 | C20H32O10 | +1.6 | 385[M-H- CH2O2] 223[M-H- C11H12O4] 205[M-H- C11H14O5] 153[M-H- C10H14O9] 138[M-H- C11H17O9] 113[M-H- C14H22O8] | 2-({6-O-[(2E)-6-Hydroxy-2,6-dimethyl-2,7-octadienoyl]-β-D-glucopyranosyl}oxy)-2-methylpropanoic acid | 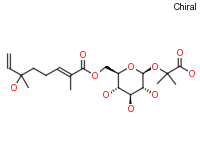 |
| 41 | 7.172 | 387.1655 | C_18_H_28_O_9_ | -1.8 | 207[M-H-C_6_H_12_O_6_]^-^163[M-H-C_12_H_16_O_4_]^-^ 135[M-H-C_13_H_16_O_6_]^-^ | {(1R,2R)-2-[(2Z)-5-(β-D-glucopyranosyloxy)pent-2-en-1-yl]-3-oxocyclopentyl}acetic acid | 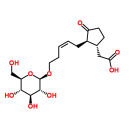 |
| 42 | 7.350 | 209.0814 | C_11_H_14_O_4_ | -7.2 | 194[M-H-CH_3_]^-^179[M-H-C_2_H_6_]^-^161[M-H-C_2_H_8_O_5_]^-^151[M-H-C_3_H_6_O]^-^  133[M-H-C_4_H_12_O]^-^ 121[M-H-C_5_H_12_O]^-^ | Sinapyl alcohlo | 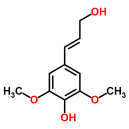 |
| 43 | 7.350 | 415.1604 | C19H28O10 | -5.8 | 269[M-H- C6H10O4] 207[M-H- C7H12O7] 161[M-H- C13H18O5] 143[M-H- C13H20O6] 125[M-H- C13H22O7] 113[M-H- C13H18O8] 101[M-H- C13H14O9] 85[M-H- C15H22O8] | Benzyl 6-O-(6-deoxy-α-L-mannopyranosyl)-β-D-glucopyranoside | 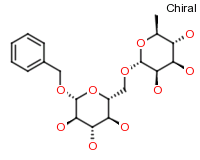 |
| 44 | 7.361 | 337.0923 | C_16_H_18_O_8_ | -3.9 | 191[M-H-C_9_H_6_O_2_]^-^173[M-H-C_9_H_8_O_3_]^-^ 163[M-H-C_7_H_10_O_5_]^-^145[M-H-C_7_H_12_O_6_]^-^ 119[M-H-C_8_H_10_O_7_]^-^ | p-Coumaroyl quinic acid | 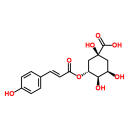 |
| 45 | 7.466 | 551.1765 | C26H32O13 | 2.0 | 389[M-H-C6H10O5]-359[M-H-C10H8O4]-341 [M-H-C10H10O5]-282[M-H-C12H13O7]- 193[M-H-C16H22O9]- 150[M-H-C18H25O10]- 134[M-H- C18H25O11]- | (2E)-3-[4-({(1S,2S)-1-[4-(β-D-Glucopyranosyloxy)-3-methoxyphenyl]-1,3-dihydroxy-2-propanyl}oxy)-3-methoxyphenyl]acrylic acid | 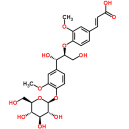 |
| 46 | 7.435 | 537.1972 | C_26_H_34_O_12_ | -3.4 | 375M-H-C_6_H_10_O_5_]^-^ 327[M-H-C_7_H_14_O_7_]^-^ 312[M-H-C_7_H_14_O_8_]^-^ 195[M-H-C_16_H_22_O_8_]^-^ 180[M-H-C_17_H_25_O_8_]^-^ 179[M-H-C_16_H_22_O_9_]^-^ 165 [M-H-C_17_H_24_O_9_]^-^ 164[M-H-C_17_H_25_O_9_]^-^ 150 [M-H-C_17_H_22_O_10_]^-^ 122 [M-H-C_19_H_27_O_10_]^-^ | (2E)-3-(4-{[1,3-Dihydroxy-1-(4-hydroxy-3-methoxyphenyl)-2-propanyl]oxy}-3-methoxyphenyl)-2-propen-1-yl β-D-glucopyranoside | 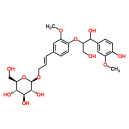 |
| 47 | 7.508 | 509.2234 | C22H38O13 | -0.4 | 463[M-H- CH_2_O_2_] 331[]M-H- C_6_H_10_O_6_] 215[M-H- C_13_H_25_O_7_] 187[M-H- C_15_H_29_O_7_] 161[M-H- C_16_H_28_O_8_] 132[M-H- C_17_H_29_O_9_] | 1-O-[(2E)-8-(β-D-Glucopyranosyloxy)-2,6-dimethyl-2-octenoyl]-β-D-glucopyranose | 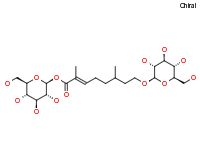 |
| 48 | 7.519 | 585.2183 | C27H38O14 | 0.0 | 329[M-H- C_12_H_16_O_6_] 314[M-H- C_12_H_15_O_7_] 195[M-H- C_16_H_22_O_11_] 165[M-H- C_21_H_24_O_9_] 150[M-H- C_18_H_27_O_12_] | Methyl (1R,4aS,6S,7R,7aS)-6-({[(2S,3R,4S)-2-(β-D-glucopyranosyloxy)-4-(2-hydroxyethyl)-3-vinyl-3,4-dihydro-2H-pyran-5-yl]carbonyl}oxy)-1-hydroxy-7-methyl-1,4a,5,6,7,7a-hexahydrocyclopenta[c]pyran-4 -carboxylate | 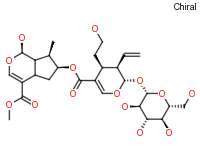 |
| 49 | 7.540 | 463.2179 | C21H36O11 | -1.3 | 331[M-H- C_5_H_8_O_4_] 285[M-H- C_7_H_14_O_5_] 161[M-H- C_15_H_26_O_6_] 113[M-H- C_16_H_30_O_8_] 101[M-H- C_17_H_30_O_8_] 85[M-H- C_17_H_30_O_9_] 71[M-H- C_18_H_32_O_9_] | 2-Hydroxy-5-isopropenyl-2-methylcyclohexyl 6-O-[3,4-dihydroxy-4-(hydroxymethyl)tetrahydro-2-furanyl]-β-D-glucopyranoside | 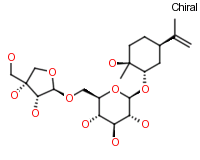    p-薄荷-8-烯-1,2-二醇2-O-芹糖基-(1→6)-吡喃葡萄糖苷 |
| 50 | 7.561 | 681.2395 | C32H42O16 | -2.2 | 519M-H-C_6_H_10_O_5_]^-^ 357[M-H-C_12_H_20_O_10_]^-^ 342[M-H-C_13_H_23_O_10_]^-^ 327[M-H-C_14_H_26_O_10_]^-^ 191[M-H-C_21_H_30_O_13_]^-^ 151[M-H-C_24_H_34_O_13_]^-^ 136[M-H-C_25_H_37_O_13_]^-^ | 4-{(1S,3aR,4S,6aR)-4-[4-(β-D-Glucopyranosyloxy)-3-methoxyphenyl]tetrahydro-1H,3H-furo[3,4-c]furan-1-yl}-2-methoxyphenyl β-D-glucopyranoside | 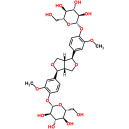 |
| 51 | 7.582 | 515.1190 | C25H24O12 | -1.9 | 353[M-H-C_9_H_6_O_3_]^-^191[M-H-C_18_H_12_O_6_]^-^179[M-H-C_16_H_16_O_8_]^-^173[M-H-C_18_H_14_O_7_]^-^  135[M-H-C_17_H_16_O_10_]^-^ | Dicaffeoylquinic acid glucoside |  |
| 52 | 7.625 | 717.2500 | C33H44O17 | -1.1 | 549M-H-C_6_H_10_O_5_]^-^ 387[M-H-C_12_H_20_O_10_]^-^ 372[M-H-C_13_H_23_O_10_]^-^ 357[M-H-C_14_H_26_O_10_]^-^ 191[M-H-C_22_H_32_O_14_]^-^ 181[M-H-C_24_H_34_O_13_]^-^166[M-H-C_25_H_37_O_13_]^-^ 151[M-H-C_25_H_36_O_14_]^-^136[M-H-C_26_H_39_O_14_]^-^ | 4-{4-[4-(β-D-Glucopyranosyloxy)-3,5-dimethoxyphenyl]tetrahydro-1H,3H-furo[3,4-c]furan-1-yl}-2-methoxyphenyl β-D-glucopyranoside | 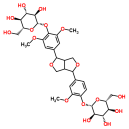 |
| 53 | 7.665 | 537.1972 | C26H34O12 | -1.9 | 375M-H-C_6_H_10_O_5_]^-^ 327[M-H-C_7_H_14_O_7_]^-^ 312[M-H-C_7_H_14_O_8_]^-^ 195[M-H-C_16_H_22_O_8_]^-^ 180[M-H-C_17_H_25_O_8_]^-^ 179[M-H-C_16_H_22_O_9_]^-^ 165 [M-H-C_17_H_24_O_9_]^-^ 164[M-H-C_17_H_25_O_9_]^-^ 150 [M-H-C_17_H_22_O_10_]^-^ 122 [M-H-C_19_H_27_O_10_]^-^ | 4-(1,3-Dihydroxy-2-{4-[(1E)-3-hydroxy-1-propen-1-yl]-2-methoxyphenoxy}propyl)-2-methoxyphenyl hexopyranoside | 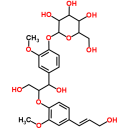 |
| 54 | 7.676 | 539.2129 | C26H36O12 | -1.3 | 491M-H-CH_4_O_2_]^-^ 377M-H-C_6_H_10_O_5_]^-^ 329[M-H-C_7_H_14_O_7_]^-^ 314 [M-H-C_7_H_14_O_8_]^-^ 195[M-H-C_16_H_24_O_8_]^-^181[M-H-C_16_H_22_O_9_]^-^180[M-H-C_17_H_27_O_8_]^-^166[M-H-C_17_H_25_O_9_]^-^ 165 [M-H-C_17_H_26_O_9_]^-^ 150 [M-H-C_17_H_25_O_10_]^-^ 122 [M-H-C_19_H_29_O_10_]^-^ | 4-{(1S,2R)-1,3-Dihydroxy-2-[4-(3-hydroxypropyl)-2-methoxyphenoxy]propyl}-2-methoxyphenyl β-D-glucopyranoside | 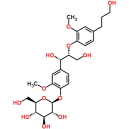 |
| 55 | 7.697 | 367.1029 | C17H20O9 | -4.4 | 193[M-H-C_7_H_10_O_5_]^-^191[M-H-C_10_H_8_O_3_]^-^173[M-H- C_10_H_10_O_4_]^-^ 134[M-H- C_9_H_13_O_7_]^-^ | 3-O-caffeoyl Feruloylquinic Acid | 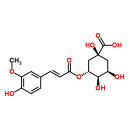 |
| 56 | 7.697 | 209.0814 | C11H14O4 | -9.6 | 165[M-H-CO2]147[M-H-CO3]91[M-H-C5H10O3] | Niduloic acid | 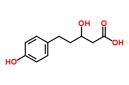 |
| 57 | 7.729 | 741.2606 | C34H46O18 | -0.5 | 417[M-H-C_12_H_20_O_10_]^-^402[M-H-C_13_H_23_O_10_]^-^387[M-H-C_14_H_26_O_10_] | Eleutheroside E | 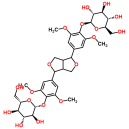 |
| 58 | 7.814 | 539.2129 | C26H36O12 | -3.2 | 491M-H-CH_4_O_2_]^-^ 377M-H-C_6_H_10_O_5_]^-^ 329[M-H-C_7_H_14_O_7_]^-^ 314 [M-H-C_7_H_14_O_8_]^-^ 195[M-H-C_16_H_24_O_8_]^-^181[M-H-C_16_H_22_O_9_]^-^180[M-H-C_17_H_27_O_8_]^-^166[M-H-C_17_H_25_O_9_]^-^ 165 [M-H-C_17_H_26_O_9_]^-^ 150 [M-H-C_17_H_25_O_10_]^-^ 122 [M-H-C_19_H_29_O_10_]^-^ | (1S,2S)-3-Hydroxy-1-(4-hydroxy-3-methoxyphenyl)-2-[4-(3-hydroxypropyl)-2-methoxyphenoxy]propyl β-D-glucopyranoside | 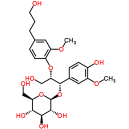 |
| 59 | 7.940 | 595.1299 | C26H28O16 | -0.3 | 300[M-H- C_11_H_19_O_9_] 271[M-H- C_11_H_16_O_11_] 255[M-H- C_15_H_16_O_9_] 243[M-H- C_15_H_12_O_10_] 151[M-H- C_21_H_16_O_11_] | Quercetin-3-arabinoglucoside | 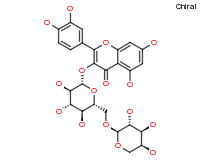 |
| 60 | 7.918 | 519.2078 | C23H36O13 | +0.8 | 361[M-H- C7H10O4] 346[M-H- C8H13O4] 329[M-H- C7H10O6] 315[M-H- C8H12O6] 285[M-H- C9H14O7] 191[M-H- C16H24O7] 161[M-H- C17H26O8] | 7-(Acetoxymethyl)-4-[(β-D-glucopyranosyloxy)methyl]-6,7-dihydroxy-1,4a,5,6,7,7a-hexahydrocyclopenta[c]pyran-1-yl 3-methylbutanoate | 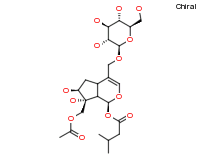 |
| 61 | 8.529 | 581.2234 | C28H38O13 | -1.7 | 419[M-H-C_6_H_10_O_5_]^-^ 404[M-H-C_7_H_13_O_5_]^-^389[M-H-C_8_H_16_O_5_]^-^ 222[M-H-C_16_H_22_O_9_]^-^ 208[M-H-C_17_H_25_O_9_]^-^190[M-H-C_17_H_27_O_10_]175[M-H-C_18_H_30_O_10_]^-^ | 4-{[(3R,4R,5S)-5-(4-Hydroxy-3,5-dimethoxyphenyl)-4-(hydroxymethyl)tetrahydro-3-furanyl]methyl}-2,6-dimethoxyphenylβ-D-glucopyranoside | 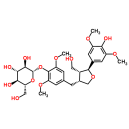 |
| 62 | 8.118 | 371.1342 | C17H24O9 | -2.4 | 285[M-H- C4H6O2] 241[M-H- C5H6O4] 197[M-H- C7H10O5] 191[M-H- C7H16O5] C6H10O4 [M-H- C11H13O5]  101[M-H- C12H14O7] | Methyl2-O-β-D-glucosyloxy-4-methoxybenzenepropanoate | 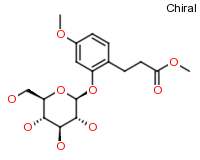 |
| 63 | 8.129 | 463.2179 | C22H24O11 | 1.5 | 301[M-H- C_6_H_10_O_5_] 285[M-H- C_7_H_14_O_5_] 191[M-H- C_12_H_16_O_7_] 149[M-H- C_13_H_14_O_9_] 125[M-H- C_16_H_18_O_8_] 101[M-H- C_17_H_14_O_9_] | Hesperetin 7-O-glucoside | 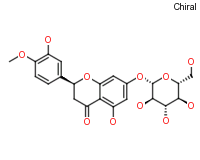 |
| 64 | 8.412 | 609.1456 | C27H30O16 | -0.3 | 300[M-H- C12H21O9] 271[M-H- C15H14O9] 255[M-H- C15H14O10] 243[M-H- C16H14O10] 227[M-H- C17H18O10] 151[M-H- C22H18O11] | Rutin | 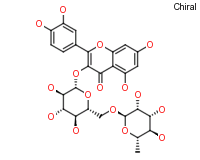 |
| 65 | 8.412 | 567.2078 | C27H36O13 | -3.0 | 341[M-H- C_12_H_18_O_4_] 314[M-H- C_13_H_17_O_5_] 311[M-H- C_12_H_16_O_6_] 269[M-H- C_14_H_18_O_7_] | 4-(1,3-Dihydroxy-2-{4-[(1E)-3-hydroxy-1-propen-1-yl]-2,6-dimethoxyphenoxy}propyl)-2-methoxyphenyl β-D-allopyranoside | 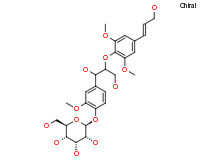 |
| 66 | 8.443 | 521.2023 | C26H34O11 | 0.0 | 329[M-H- C_7_H_12_O_6_]^-^418[M-H-C_7_H_13_O_5_]^-^403[M-H-C_8_H_16_O_5_]^-^ | [7-Hydroxy-1-(4-hydroxy-3-methoxyphenyl)-3-(hydroxymethyl)-6-methoxy-1,2,3,4-tetrahydro-2-naphthalenyl]methylβ-D-glucopyranoside | 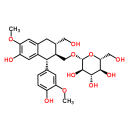 |
| 67 | 8.612 | 463.0877 | C21H20O12 | -0.9 | 300[M-H- C6H11O5] 271[M-H- C6H8O7] 255[M-H- C9H4O6] 243[M-H- C10H4O6] 179[M-H- C15H8O6] 151[M-H- C16H8O7] | Orientin/  hyperin | 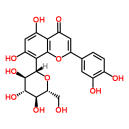  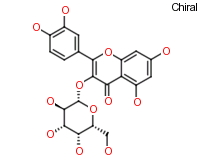 |
| 68 | 8.623 | 449.1084 | C21H22O11 | -2.4 | 287[M-H- C_6_H_10_O_5_] 269[M-H- C_6_H_12_O_6_] 151[M-H- C_16_H_10_O_6_] | Astilbin | 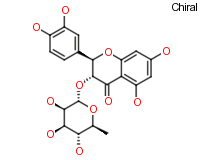 |
| 69 | 8.833 | 463.0877 | C21H20O12 | -3.2 | 300[M-H- C_6_H_11_O_5_] 271[M-H- C_6_H_8_O_7_] 255[M-H- C_9_H_4_O_6_] 243[M-H- C_10_H_4_O_6_] 179[M-H- C_15_H_8_O_6_] 151[M-H- C_16_H_8_O_7_] | Orientin/  hyperin | 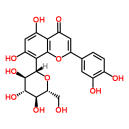  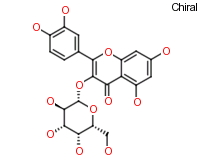 |
| 70 | 9.054 | 441.1761 | C21H30O10 | -1.8 | 327[M-H- C9H6] 307[M-H- C9H10O] 247[M-H- C7H14O6] 191[M-H- C14H18O4] 163[M-H- C15H18O5] 133[M-H- C12H20O9] 125[M-H- C15H24O7] | (2E)-3-Phenyl-2-propen-1-yl 6-O-(6-deoxy-α-L-mannopyranosyl)-β-D-glucopyranoside | 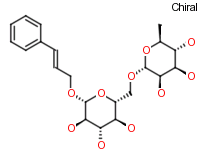 |
| 71 | 9.380 | 549.1972 | C27H34O12 | 2.5 | 387[M-H-C6H10O5]-372[M-H-C7H10O8]357[M-H-C8H10O11]191[M-H-C16H22O9]181[M-H-C18H24O8]166[M-H-C19H27O8]151[M-H-C19H26O9]136[M-H-C20H29O9]- | Medioresinol 4'-O-β-D-glucopyranoside | 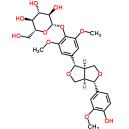 |
| 72 | 9.433 | 515.1190 | C25H24O12 | -1.0 | 353[M-H-C_9_H_6_O_3_]^-^191[M-H-C_18_H_12_O_6_]^-^179[M-H-C_16_H_16_O_8_]^-^173[M-H-C_18_H_14_O_7_]^-^  135[M-H-C_17_H_16_O_10_]^-^ | 3,4-Dicaffeoyl-quinic acid | 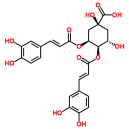 |
| 73 | 9.507 | 519.1866 | C_26_H_32_O_11_ | -0.4 | 357[M-H-C_6_H_10_O_5_]^-^ 342[M-H-C_7_H_13_O_5_]^-^ 327[M-H-C_8_H_16_O_5_]^-^ 191[M-H-C_15_H_28_O_8_]^-^ 151[M-H-C_18_H_24_O_8_]^-^ 136[M-H-C_19_H_27_O_8_]^-^ | 4-[4-(4-Hydroxy-3-methoxyphenyl)tetrahydro-1H,3H-furo[3,4-c]furan-1-yl]-2-methoxyphenyl β-D-glucopyranoside | 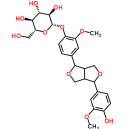 |
| 74 | 9.801 | 579.2078 | C28H36O13 | -2.9 | 417[M-H-C_6_H_10_O_5_]^-^402[M-H-C_7_H_13_O_5_]^-^387[M-H-C_8_H_16_O_5_]^-^ | Syringaresinol monoglucoside | 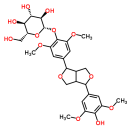 |
| 75 | 9.927 | 515.1190 | C25H24O12 | -2.9 | 353[M-H-C_9_H_6_O_3_]^-^191[M-H-C_18_H_12_O_6_]^-^179[M-H-C_16_H_16_O_8_]^-^173[M-H-C_18_H_14_O_7_]^-^  135[M-H-C_17_H_16_O_10_]^-^ | 3,5-Dicaffeoyl-quinic acid | 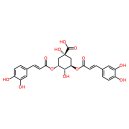 |
| 76 | 10.033 | 447.0927 | C21H20O11 | 0.0 | 300[M-H- C_6_H_11_O_4_] 285[M-H- C_6_H_10_O_5_] 271[M-H- C_6_H_8_O_6_] 255[M-H- C_10_H_8_O_4_] 243[M-H- C_8_H_12_O_6_] 227[M-H- C_11_H_8_O_5_] 151[M-H- C_16_H_8_O_6_] | Quercitrin | 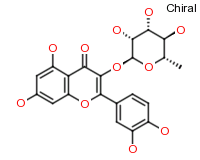 |
| 77 | 10.148 | 563.1765 | C27H32O13 | -2.3 | 337[M-H-C11H14O5]325[M-H- C8H14O8]307[M-H-C12H16O6]295[M-H-C9H16O9]267[M-H-C14H16O7]251[M-H- C15H20O7] | Methyl (1S,4aS,5R,7aS)-1-(β-D-glucopyranosyloxy)-5-{[(2E)-3-(4-hydroxyphenyl)-2-propenoyl]oxy}-7-(methoxymethyl)-1,4a,5,7a-tetrahydrocyclopenta[c]pyran-4-carboxylate | 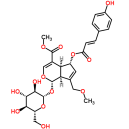 |
| 78 | 10.275 | 601.1193 | C28H26O15 | +2.0 | 395[M-H- C_10_H_6_O_5_] 353[M-H- C_12_H_8_O_6_] 335[M-H- C_12_H_10_O_7_] 233[M-H- C_19_H_12_O_8_] 191[M-H- C_21_H_14_O_9_] 179[M-H- C_19_H_18_O_11_] 173[M-H- C_21_H_16_O_10_] 161[M-H- C_19_H_20_O_12_] 135[M-H- C_20_H_18_O_13_] | 1-Methoxyoxalyl-3,5-dicaffeoylquinic acid | 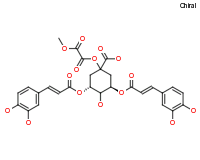 |
| 79 | 10.316 | 433.1135 | C21H22O10 | -0.9 | 271[M-H- C_9_H_6_O_3_] 177[M-H- C_12_H_16_O_6_] 151[M-H- C_14_H_18_O_6_] 119[M-H-C_16_H_10_O_7_] 107[M-H- C_15_H_18_O_8_] | 2-O-Caffeoyl arbutin | 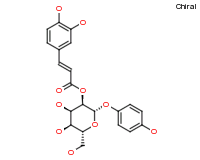 |
| 80 | 10.454 | 187.0970 | C9H16O4 | -8.6 | 169[M-H- H_2_O] 143[M-H- CO_2_] 125[M-H- CHO_3_] 97[M-H- C_3_H_6_O_3_] | Azelaic acid | 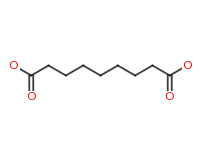 |
| 81 | 10.884 | 515.1190 | C25H24O12 | -1.0 | 353[M-H-C_9_H_6_O_3_]^-^191[M-H-C_18_H_12_O_6_]^-^179[M-H-C_16_H_16_O_8_]^-^173[M-H-C_18_H_14_O_7_]^-^  135[M-H-C_17_H_16_O_10_]^-^ | 4,5-Dicaffeoyl-quinic acid | 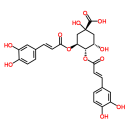 |
| 82 | 11.305 | 361.1651 | C20H26O6 | +2.5 | 346[M-H- CH_3_]^-^ 179[M-H-C_10_H_14_O_3_]^-^ 165[M-H-C_11_H_16_O_3_]^-^ | Secoisolariciresinol | 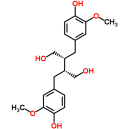 |
| 83 | 12.326 | 331.1757 | C16H28O7 | 0.0 | 285[M-H- C2H6O] 161[M-H- C10H18O2] 113[M-H- C9H14O6] 101[M-H- C10H14O6] | 2-Hydroxy-5-isopropenyl-2-methylcyclohexyl -glucopyranoside | 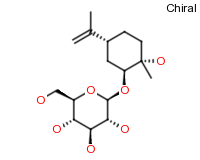 |
| 84 | 14.471 | 971.4946 | C48H76O20 | -2.4 | 647[]M-H- C_12_H_20_O_10_] 585[M-H- C_12_H_18_O_14_] 471[M-H- C_18_H_28_O_16_] | 1-O-[(3β,5ξ,18ξ)-3-{[2-O-(β-D-Galactopyranosyl)-β-D-glucopyranuronosyl]oxy}-23-hydroxy-28-oxoolean-12-en-28-yl]-β-D-glucopyranose | 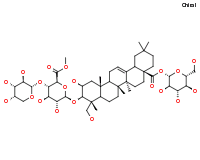 |
| 85 | 15.745 | 357.1338 | C20H2206 | -5.9 | 342[M-H-CH_3_]^-^ 327 [M-H-C_2_H_6_]^-^ 313[M-H-CO_2_]^-^221[M-H-C_8_H_8_O_2_]^-^  161[M-H- C_10_H_12_O_4_]^-^ 137[M-H-C_12_H_12_O_4_]^-^122[M-H-C_13_H_15_O_4_]^-^83[M-H-C_16_H_18_O_4_]^-^ | matairesinol | 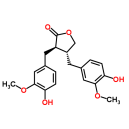 |
| 86 | 15.766 | 577.1346 | C30H26O12 | 2.4 | 385[M-H- C_10_H_8_O_4_] 311[M-H- C_12_H_10_O_7_] 193[M-H- C_20_H_16_O_8_] 178[M-H- C_21_H_19_O_8_] 163[M-H- C_21_H_18_O_9_] 149[M-H- C_21_H_16_O_10_] 134[M-H- C_22_H_19_O_10_] | 8O4/8O4-dehydrotriferulic acid | 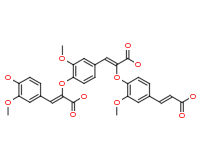 |
| 87 | 16.660 | 809.4323 | C42H66O15 | -0.7 | 647[M-H- C6H10O5] 603[M-H- C7H10O7] 471[M-H- C12H18O11] | Acanthopanaxoside E | 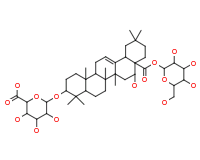 |
| 88 | 16.6860 | 955.4961 | C48H76O19 | -0.8 | 631[M-H- C_12_H_20_O_10_] 455[M-H- C_18_H_28_O_16_] | Acanjaposide G/Acanjaposide H | 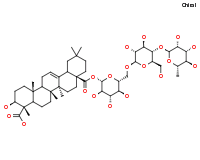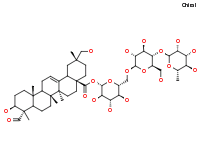 |
| 89 | 17.007 | 331.2484 | C18H36O5 | -3.9 | 313[M-H- H_2_O] 295[M-H- H_4_O_2_] 157[M-H- C_9_H_18_O_3_] 127[M-H- C_11_H_24_O_3_] | Trihydroxyoctadecanoicacid | 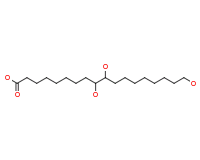 |
| 90 | 17.038 | 763.4327 | C41H64O13 | -4.8 | 719[M-H- CO_2_] 617[M-H- C_5_H_6_O_5_] 573[M-H- C_6_H_6_O_7_] 555[M-H- C_6_H_8_O_8_] 485[M-H- C_10_H_14_O_9_] 441[M-H- C_11_H_14_O_11_] | 28-Hydroxy-28-oxoolean-12-en-3-yl 3-O- xylopyranosyl- glucopyranosiduronic acid | 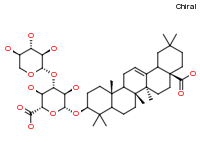 |
| 91 | 17.164 | 1203.6315 | C63H96O22 | -0.2 | 733[M-H- C_22_H_30_O_11_] 469[M-H- C_35_H_58_O_16_] 367[M-H- C_44_H_68_O_15_] 323[M-H- C_42_H_72_O_19_] | 3-{[2,6-Dideoxy-3-O-methyl-ribo-hexopyranosyl-(1->4)-2,6-dideoxy-3-O-methyl-arabino-hexopyranosyl-(1->4)-2,6-dideoxy-3-O-methyl-β-D-arabino-hexopyranosyl -(1->4)-2,6-dideoxy-3-O-methyl-β-D-ribo-hexopyranosyl-(1->4)-2,6-dideoxy-3-O-methyl-β-D-ribo-hexopyranosyl]oxy}-8,14,17-trihydroxy-20-oxopregn-5-en-12-yl benzoate | 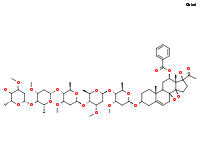 |
| 92 | 17.175 | 329.2328 | C18H34O5 | -3.3 | 311[M-H-H_2_O]^-^293[M-H-H_4_O_2_]^-^195[M-H-C_21_H_26_O_7_]^-^165[M-H-C_21_H_24_O_9_]^-^ | trihydroxy-10(11)-octadecenoic acid | 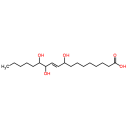 |
| 93 | 17.238 | 807.4147 | C42H64O15 | +1.2 | 645[M-H- C_6_H_10_O_5_] 583[M-H- C_6_H_8_O_9_] 469[M-H- C_12_H_18_O_11_] | 3-O-β-D-Glucuronopyranosyl gypsogenin 28-O-β-D-glucopyranoside | 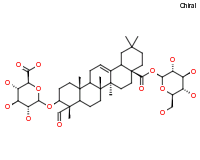 |
| 94 | 17.270 | 749.4476 | C42H64O15 | +2.0 | 603 [M-H- C6H10O4] 585 [M-H- C6H12O5] 471 [M-H-C11H18O8] | α-HEDERIN | 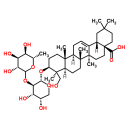 |
| 95 | 17.470 | 1245.6421 | C65H98O23 | -1.4 | 775[M-H- C_26_H_30_O_8_] 733[M-H- C_24_H_32_O_12_] 299[M-H- C_51_H_78_O_16_] | 3-{[4-O-Acetyl-2,6-dideoxy-3-O-methyl-β-D-arabino-hexopyranosyl-(1->4)-2,6-dideoxy-3-O-methyl-β-D-arabino-hexopyranosyl-(1->4)-2,6-dideoxy-3-O-methyl-β-D-arabino -hexopyranosyl-(1->4)-2,6-dideoxy-3-O-methyl-β-D-ribo-hexopyranosyl-(1->4)-2,6-dideoxy-3-O-methyl-β-D-ribo-hexopyranosyl]oxy}-8,14,17-trihydroxy-20-oxopregn-5-en-12-yl benzoate | 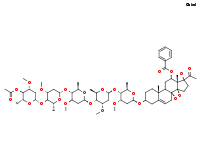 |
| 96 | 17.585 | 603.3897 | C35H56O8 | -2.0 | 449[M-H-C4H8O3]308[M-H-C12H23O8] | Hederagenin 3-O-arabinoside | 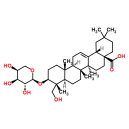 |
| 97 | 17.617 | 793.4374 | C42H66O14 | -2.9 | 631[M-H- C6H10O5] 569[M-H- C6H8O9] 455[M-H- C12H18O11] | Silphioside G | 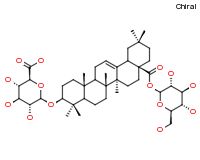 |
| 98 | 18.468 | 647.3795 | C36H56O10 | -0.9 | 603[M-H- CO2] 571[M-H- C2H4O3] 471[M-H- C6H8O6] 439[M-H- C6H8O8] | hederagenin-3-o -glucuronopyraoside | 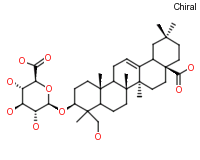 |
| 99 | 19.015 | 717.4214 | C40H62O11 | +1.3 | 571[M-H- C_6_H_10_O_4_] 554[M-H- C_6_H_11_O_5_] 439[M-H- C_11_H_18_O_8_] | Ciwujianoside E | 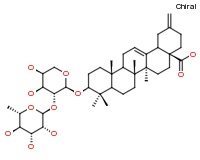 |
| 100 | 19.153 | 749.4476 | C41H66O12 | +0.8 | 587[M-H- C_6_H_9_O_5_] 569[M-H- C_5_H_7_O_7_] 455[M-H- C_11_H_17_O_9_] | 3-{[2-O-(Lyxopyranosyl)-galactopyranosyl]oxy}olean-12-en-28-oic acid | 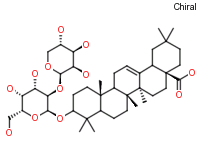 |
| 101 | 19.415 | 631.3846 | C36H56O9 | +0.6 | 587[M-H- CO_2_] 455[M-H- C_6_H_8_O_6_] | Oleanoic acid 3-O-glucuronide | 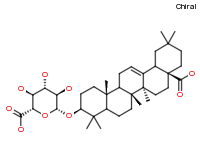 |
| 102 | 19.657 | 733.4527 | C41H66O11 | +1.6 | 587[M-H- C_6_H_10_O_4_] 569[M-H- C_6_H_12_O_5_] 455[M-H- C_11_H_18_O_8_] | Eleutheroside K |  |
| 103 | 20.268 | 295.2273 | C18H32O3 | -5.8 | 277[M-H- H_2_O] 237[M-H- C_4_H_10_] 209[M-H-C_4_H_6_O_2_] 183[M-H- C_6_H_8_O_2_] 157[M-H- C_9_H_14_O] | 13-Hydroxy-9,11-octadecadienoic acid |  |
| 104 | 21.814 | 271.2273 | C16H32O3 | -1.8 | 225[M-H- CH_2_O_2_] | 3-Hydroxyhexadecanoic acid |  |

**TableS2:** Specific information of biomarkers in the blood of ovariectomized osteoporosis model rats.

| **NO** | **Rt-m/z** | **Error ppm** | **Ion**  **form** | **Proposed**  **Composition** | **Postulated Identity** | **Trend**  **In ovx** |
| --- | --- | --- | --- | --- | --- | --- |
| **1** | 0.70_116.0704 | -1.7 | [M+H]^+^ | C_5_H_9_NO_2_ | L-Proline | ↑ |
| **2** | 1.31_204.1223 | -3.5 | [M+H]^+^ | C_9_H_17_NO_4_ | L-Acetylcarnitine | ↓ |
| **3** | 1.58_167.0215 | +2.7 | [M-H]^-^ | C_5_H_4_N_4_O_3_ | Uric acid | ↓ |
| **4** | 1.75_180.0670 | +2.0 | [M-H]^-^ | C_9_H_11_NO_3_ | L-Tyrosine | ↑ |
| **5** | 3.11_203.0828 | +1.1 | [M-H]^-^ | C_11_H_12_N_2_O_2_ | L-Tryptophan | ↓ |
| **6** | 4.48_516.2964 | -4.9 | [M+H]^+^ | C_26_H_45_NO_7_S | Taurocholic acid | ↓ |
| **7** | 7.48_391.2114 | -3.6 | [M+FA-H]^-^ | C_21_H_30_O_4_ | Corticosterone | ↓ |
| **8** | 7.69_448.3052 | -3.7 | [M-H]^-^ | C_26_H_43_NO_5_ | Chenodeoxycholic acid glycine conjugate | ↑ |
| **9** | 7.98_453.2840 | -4.3 | [M+FA-H]^-^ | C_24_H_40_O_5_ | Cholic acid | ↑ |
| **10** | 8.19_349.2365 | -5.1 | [M-H]^-^ | C_20_H_32_O_2_ | Arachidonic acid | ↓ |
| **11** | 8.76_315.2297 | +0.8 | [M+Na]^+^ | C_19_H_32_O_2_ | 5a-Androstane-3b,17b-diol | ↓ |
| **12** | 9.40_313.2372 | -4.0 | [M-H]^-^ | C_18_H_34_O_4_ | 9,10-DHOME | ↓ |
| **13** | 9.95_568.3356 | -3.2 | [M+Na]^+^ | C_28_H_52_NO_7_P | LysoPC(22:6(4Z,7Z,10Z,13Z,16Z,19Z)) | ↓ |
| **14** | 10.85_319.2269 | -2.9 | [M-H]^-^ | C_20_H_32_O_3_ | 5-HETE | ↓ |
| **15** | 10.86_303.2306 | +4.0 | [M+H]^+^ | C_20_H_30_O_2_ | Linoleic acid | ↓ |
| **16** | 12.43_826.5387 | +0.7 | [M+H]^+^ | C_48_H_76_NO_8_P | PC(22:6(4Z,7Z,10Z,13Z,16Z,19Z)/18:4(6Z,9Z,12Z,15Z)) | ↓ |

**↑:**Compared with the sham operation group, the blood metabolites in the OVX model group increased significantly.

**↓:**Compared with the sham operation group, the blood metabolites in the OVX model group were significantly reduced.

**TableS3:** Secondary fragmentation of potential biomarkers in the blood of ovariectomized osteoporosis model rats

| **NO** | **Rt_m/z** | **Proposed**  **Composition** | **Postulated Identity** | **MS/MS** |
| --- | --- | --- | --- | --- |
| **1** | 0.70_116.0704 | C_5_H_9_NO_2_ | L-Proline | 116[M+H]^+^  70[M+H-HCOOH]^+^ |
| **2** | 1.31_204.1223 | C_9_H_17_NO_4_ | L-Acetylcarnitine | 204[M+H]^+^  144[M+H-C_2_H_4_O_2_]^+^ |
| **3** | 1.58_167.0215 | C_5_H_4_N_4_O_3_ | Uric acid | 167[M-H]^-^  124[M-H-CHNO]^-^  96[M-H-C_2_HNO_2_]^-^ |
| **4** | 1.75_180.0670 | C_9_H_11_NO_3_ | L-Tyrosine | 180[M-H]^-^  163[M-H-NH_3_]^-^  134[M-H-C_2_H_6_O]^-^ |
| **5** | 3.11_203.0828 | C_11_H_12_N_2_O_2_ | L-Tryptophan | 203[M-H]^-^  186[M-H-NH_3_]^-^  142[M-H-CH_3_NO_2_]^-^ |
| **6** | 4.48_516.2964 | C_26_H_45_NO_7_S | Taurocholic acid | 453[M+H]^+^  435[M+H-H_2_O]^+^  417[M+H-H_4_O_2_]^+^  307[M+H-H_14_C_7_NSO_4_]^+^ |
| **7** | 7.48_391.2114 | C_21_H_30_O_4_ | Corticosterone | 391[M+FA-H]^-^  373[M+FA-H_2_O]^-^  355[M+FA-2H_2_O]^-^  331[M-H-C_2_H_4_O_2_]^-^ |
| **8** | 7.69_448.3052 | C_26_H_43_NO_5_ | Chenodeoxycholic acid glycine conjugate | 448[M-H]^-^  430[M-H-H_2_O]^-^  402[M-H-H_2_CO_2_]^-^  291[M-H-H_11_C_7_NO_3_]^-^ |
| **9** | 7.98_453.2840 | C_24_H_40_O_5_ | Cholic acid | 453[M+FA-H]^-^  435[M+FA-H_2_O]^-^  407[M+FA-H_2_CO_2_]^-^  351[M+FA-H_10_C_5_O_2_]^-^ |
| **10** | 8.19_349.2365 | C_20_H_32_O_2_ | Arachidonic acid | 349[M+FA-H]^-^  331[M+FA-H_2_O]^-^  303[M+FA-H_2_CO_2_]^-^ |
| **11** | 8.76_315.2297 | C_19_H_32_O_2_ | 5a-Androstane-3b,17b-diol | 315[M+Na]^+^  297[M+Na-H_2_O]^+^  279[M+Na-2H_2_O]^+^ |
| **12** | 9.40_313.2372 | C_18_H_34_O_4_ | 9,10-DHOME | 313[M-H]^-^  295[M-H-H_2_O]^-^  277[M-H-H_2_O-H_2_O]^-^  183[M-H-C_7_H_14_O_2_]^-^ |
| **13** | 9.95_568.3356 | C_28_H_52_NO_7_P | LysoPC(20:3(8Z,11Z,14Z)) | 568[M+Na]^+^  550[M+Na-H_2_O]^+^  508[M+H-C_3_H_10_N]^+^ |
| **14** | 10.85_319.2269 | C_20_H_32_O_3_ | 5-HETE | 319[M-H]^-^  301[M-H-H_2_O]^-^  275[M-H-HCOOH]^-^  257[M-H-CH_4_O_3_]^-^ |
| **15** | 10.86_303.2306 | C_20_H_30_O_2_ | Linoleic acid | 303[M+Na]^+^  285[M+Na-H_2_O]^+^  243[M+Na-C_2_H_4_O_2_]^+^  187[M+Na-C_7_H_16_O]^+^ |
| **16** | 12.43_826.5387 | C_48_H_76_NO_8_P | PC(22:6(4Z,7Z,10Z,13Z,16Z,19Z)/18:4(6Z,9Z,12Z,15Z)) | 826[M+H]^+^  184[M+H-C_43_H_62_O_4_]^+^ |

**TableS4: Table of potential biomarkers in blood of ASSL and NYL intervention model rats**

| **NO** | **Rt-M/Z** | **Proposed**  **Composition** | **Postulated Identity** | **Trend**  **In ovx** | **NYL** | **ASSLH** | **ASSLM** | **ASSLL** |
| --- | --- | --- | --- | --- | --- | --- | --- | --- |
| **1** | 0.70_116.0704 | C_5_H_9_NO_2_ | L-Proline | ↑ | +** | +** | +** | +** |
| **2** | 1.31_204.1223 | C_9_H_17_NO_4_ | L-Acetylcarnitine | ↓ | + | + | + | - |
| **3** | 1.58_167.0215 | C_5_H_4_N_4_O_3_ | Uric acid | ↓ | +* | +* | +* | + |
| **4** | 1.75_180.0670 | C_9_H_11_NO_3_ | L-Tyrosine | ↑ | +** | +** | +* | + |
| **5** | 3.11_203.0828 | C_11_H_12_N_2_O_2_ | L-Tryptophan | ↓ | - | - | - | - |
| **6** | 4.48_516.2964 | C_26_H_45_NO_7_S | Taurocholic acid | ↓ | + | + | + | + |
| **7** | 7.48_391.2114 | C_21_H_30_O_4_ | Corticosterone | ↓ | - | - | - | - |
| **8** | 7.69_448.3052 | C_26_H_43_NO_5_ | Chenodeoxycholic acid glycine conjugate | ↑ | +** | +** | +* | + |
| **9** | 7.98_453.2840 | C_24_H_40_O_5_ | Cholic acid | ↑ | +* | +** | +* | +* |
| **10** | 8.19_349.2365 | C_20_H_32_O_2_ | Arachidonic acid | ↓ | - | - | - | - |
| **11** | 8.76_315.2297 | C_19_H_32_O_2_ | 5a-Androstane-3b,17b-diol | ↓ | - | - | - | - |
| **12** | 9.40_313.2372 | C_18_H_34_O_4_ | 9,10-DHOME | ↓ | - | - | - | - |
| **13** | 9.95_568.3356 | C_28_H_52_NO_7_P | LysoPC(22:6(4Z,7Z,10Z,13Z,16Z,19Z)) | ↓ | +** | +** | +** | + |
| **14** | 10.85_319.2269 | C_20_H_32_O_3_ | 5-HETE | ↓ | + | +** | + | + |
| **15** | 10.86_303.2306 | C_20_H_30_O_2_ | Linoleic acid | ↓ | + | + | + | + |
| **16** | 12.43_826.5387 | C_48_H_76_NO_8_P | PC(22:6(4Z,7Z,10Z,13Z,16Z,19Z)/18:4(6Z,9Z,12Z,15Z)) | ↓ | +* | +* | +* | +** |

**↑** It indicates that the metabolite content in the blood of the OVX model group is significantly higher than that of the sham operation group;.

**↓** Represents a significant decrease in blood metabolite levels in the OVX model group compared with the sham group;

**+** Compared with the model, it showed a tendency to approach the sham operation group;

-Compared with the model, there was no tendency to approach the sham operation group

(*P<0.05;**P<0.01)
